# Genome-Wide Identification and Expression Analysis of *FD* Gene Family in Bamboos

**DOI:** 10.3390/ijms252313062

**Published:** 2024-12-05

**Authors:** Lihan Hou, Huiting Zhang, Yakun Fan, Yaling Zhang, Wengen Zhang, Guangyao Yang, Chunce Guo, Meixia Wang

**Affiliations:** 1 Jiangxi Provincial Key Laboratory of Improved Variety Breeding and Efficient Utilization of Native Tree Species, Forestry College, Jiangxi Agricultural University, Nanchang 330045, China; lihanhou@sru.edu.cn (L.H.); yakunfan0@163.com (Y.F.); zhangyaling@stu.jxau.edu.cn (Y.Z.); zhangwengen@163.com (W.Z.); yanggy2004@126.com (G.Y.); 2 Tree Fruit Research Laboratory, USDA-ARS, Wenatchee, WA 98801, USA; huiting.zhang@wsu.edu

**Keywords:** *FD* gene family, bamboo, phylogeny, gene expression patterns

## Abstract

The regulation of flowering time is a highly coordinative process that involves the interplay of multiple genes. The *FLOWERING LOCUS D* (*FD*) gene is one of those important players. In this study, we identified and characterized *FD* genes in bamboo, a plant with the unique monocarpy flowering phenomenon. An angiosperm-wide *FD* gene family analysis demonstrated that unlike the most recent common ancestor (MRCA) of angiosperms, which had only one *FD* gene, five *FD* copies were present in the MRCA of Poaceae, and the same gene copy number was retained in the MRCA of the Bambusoideae subfamily. Further analysis of the Poaceae *FD* gene family revealed five distinctive clades resulted from four duplication events, with two of these events being specific to the Bambusoideae subfamily. High levels of conservation were observed in the gene structure and amino acid composition of structural domain among the *FD* genes across bamboos and their close relatives, indicating functional conservation. Furthermore, gene expression profiling indicated that *FD* gene expression in bamboo closely resemble the expression patterns of their homologs in rice. Additionally, overexpression of two bamboo genes (*Phy.ed_05093.t1* and *Phy.ed_14669.t1*) in *Arabidopsis* resulted in an early flowering phenotype, demonstrating their involvement in the regulation of the flowering process in plants. Our findings provide a comprehensive resource for understanding the evolution, structure, expression, and function of *FD* genes in Poaceae and Bambusoideae.

## 1. Introduction

The transition from vegetative to reproductive growth holds the key to the successful continuation of a plant species. In angiosperm, this transition is realized by the initiation of flower development. Studies in the model plant *Arabidopsis thaliana* have shown that flowering is regulated by a variety of signaling pathways, including the photoperiod pathway, vernalization pathway, age pathway, and gibberellin pathway [1,2]. Different classes of flowering time genes have been identified, including those that act as signal to initiate the transition of flowering [3]. Some flowering signals, such as florigen, are mobile molecules produced in leaves and transported to the apical meristem. The molecular nature of florigen was first revealed to be a protein encoded by *Heading date 3a* (*Hd3a*) in rice, and its ortholog *FLOWERING LOCUS T* (*FT*) was identified in *Arabidopsis*. In the apical meristem, FT/Hd3a forms a protein complex with a basic leucine zipper (bZIP) transcription factor, *FD*, which activates the expression of downstream floral meristem identity genes (e.g., *APETALA1* (*AP1*), *FRUITFULL* (*FUL*), *SUPPRESSOR OF OVEREXPRESSION OF CONSTANS1* (*SOC1*)) and initiates flowering [4,5,6].

The role that *FD* plays in flowering regulation has been extensively studied in model plant species. In *Arabidopsis thaliana*, the *FD* gene (*AT4G35900*) is mainly expressed in the shoot apex meristem tissue, and its expression level increases with growth time [7]. The *atfd* mutant, in which the *FD* gene is functionally absent, exhibits a late flowering phenotype, whereas overexpression of the *FD* gene promotes flowering in plants. Additionally, overexpressing the *FT* gene in the *atfd* mutant did not restore the late flowering phenotype of the *atfd* mutant, suggesting that *FT* promotion of plant flowering is dependent on *FD* [7,8]. Paralog of *FD*, *FDP* [9] (*AT2G17770*), however, regulates genes involved in ABA signaling pathway rather than regulating flowering. In rice, Hd3a and RICE FLOWERING LOCUS T 1 (RFT1) interact with the 14-3-3 protein GF14c to form a complex that is subsequently transferred to the nucleus where it binds to the transcription factor *OsFD1* (*LOC_Os09g36910*) and forms a ternary flower-forming element activation complex (FAC). This FAC then induces *OsMADS14* gene expression, thereby initiating the flower-forming transition under both short-day and long-day conditions [10]. *OsFD4* (*LOC_Os09g36910*) shares similar properties as *OsFD1*: it forms FACs with the aforementioned flower-forming elements, and *osfd4* loss-of-function mutant plants exhibited late spike twitching [11]. Other members in the rice FD family carry out different functions. For instance, FAC containing OsFD2 protein promotes rice leaf development instead of activating flower initiation [10,12].

In Bambusoideae, Dutta et al. identified two *FD* genes, *BtFD1* and *BtFD2*, in *Bambusa tulda*. Sequence comparison showed that *BtFD1* and *BtFD2* possess divergent amino acids in the DNA binding motif, and expression analysis indicated that *BtFD1* was involved in nutritional growth and flower development, whereas *BtFD2* was low in expression under all environmental conditions and in all types of tissue [13]. A ten-fold increase in the *AtAP1* transcript level was observed in *Arabidopsis* lines overexpressing *BtFD1* compared to wild type, confirming a positive regulatory role of *BtFD1* towards flowering. However, no visible phenotype was observed in *BtFD2* overexpression *Arabidopsis* lines [13]. This signifies that the timely expression of *BtFD1* may be crucial for carrying out its programmed developmental function in plants.

Phylogenetic and expression analyses of *FD* in plants discussed above show that different members of the *FD* gene family are associated with not only flower development but also other tissue development processes, suggesting functional differentiation of members in the *FD* family. Furthermore, the *bZIP* superfamily exhibited unique expansion and evolution within angiosperms. For instance, rice encompasses a total of 89 *bZIP* genes [14]. To identify *FD*-like genes, our investigation should commence with a thorough examination of the *bZIP* gene family. The main objectives of this study were to identify *FD* genes of the bamboo subfamily, explore the evolutionary relationships of *FD* genes in the bamboo subfamily, analyze the expression patterns of *FD* genes during flowering of moso bamboo (*Phyllostachys edulis*), which is a type of woody bamboo and undergoes a singular flowering event in its lifetime, and identify and validate *FD* genes involved in flowering regulation pathways.

## 2. Results

### 2.1. Phylogenetic Analysis of the bZIP Gene Family and Identification of FD-like Genes

A total of 1333 *bZIP* genes were identified from 13 plant species, including basal angiosperms, monocots, and eudicots. Among these, monocots encompassed various major lineages of the Poaceae family, including four bamboo species. Specifically, in bamboo, we identified 179 *bZIP* genes in *P. edulis*, 97 in *Olyra latifolia*, 210 in *Bonia amplexicaulis*, and 114 in *Guadua angustifolia*. To elucidate the evolutionary relationships within the *bZIP* gene family and identify the *FD* subfamily, a maximum likelihood tree containing all the *bZIP* genes described above was constructed using RAxML. Furthermore, we categorized these genes into 10 subfamilies based on the phylogeny and the classification criteria in *Arabidopsis thaliana* [15,16] (Figure S1; Table S1). Our results revealed that subfamily A contained all the known FD proteins from model organisms—i.e., two FD family proteins in *Arabidopsis* and six in rice—thus, homologs within the same clade were denoted as *FD* genes in their respective species. A total of 55 *FD*-like genes were identified from the 13 species investigated in this study. The overall phylogenetic arrangement and classification of the *bZIP* gene family can be found in Figure S1 and Table S1.

### 2.2. Phylogenetic Relationships and Gene Structure of FD-like Genes in Angiosperms

**Figure 1:**
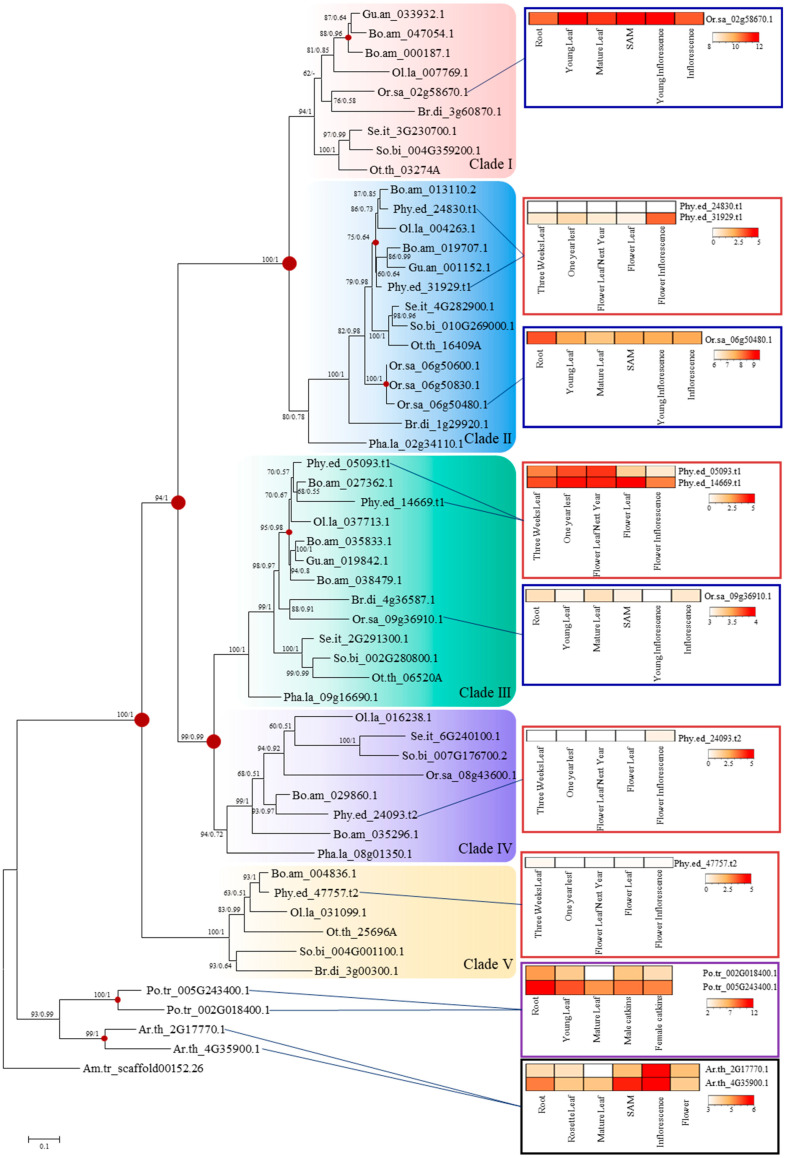
Phylogenetic tree of FD proteins and gene expression profile in each FD group. *Amborella trichopod*, *Arabidopsis thaliana*, and *Populus trichocarpa* are included as outgroups. Gene duplication events are shown on the tree with brown circles. Numbers associated with branches are ML bootstrap values and Bayesian posterior probabilities, respectively. Colored backgrounds indicate the 5 FD lineages. The color intensity in heatmaps refers to expression measurements with an RNA-seq approach in *Arabidopsis*, poplar, rice, and moso bamboo. The tissue with the highest expression was labeled in red. The full names of the abbreviations are as follows: Gu.an means *Guadua angustifolia*, Bo.am means *Bonia amplexicaulis*, Ol.la means *Olyra latifolia*, Or.sa means *Oryza sativa*, Br.di means *Brachypodium distachyon*, Se.it means *Setaria italic*, So.bi means *Sorghum bicolor*, Ot.th means *Oropetium thomaeum*, Phy.ed means *Phyllostachys edulis*, Pha.la means *Pharus latifolius*, Po.tr means *Populus trichocarpa*, Ar.th means *Arabidopsis thaliana*, Am.tr means *Amborella trichopoda*, and SAM means shoot apical meristem.

To elucidate the evolutionary relationships within the *FD* gene family in the Bambusoideae subfamily, we performed phylogenetic analyses on 55 FD proteins, identified in the previous step, using both maximum likelihood (ML) and Bayesian Inference (BI) methods and compared outcomes (Figure 1). Phylogenetic trees constructed with the two methods yielded identical tree topologies with high supporting values on each node, suggesting a robust phylogenetic relationship among the investigated sequences. It is inferred from the phylogenetic tree that one single ancestral *FD* gene was present in the common ancestor of angiosperms, and subsequent gene duplication events happened independently in monocot and dicots lineages, resulting in subgroups or clades in different lineages.

Based on the phylogeny, the *FD* genes in Poaceae were categorized into five distinct clades, namely Clade I to Clade V (Figure 1). Notably, Clade III encompassed genes from all species in the seven subfamilies of Poaceae, suggesting a high level of conservation. However, the relationships of genes within each clade did not always align with the phylogenetic relationship of the species. For instance, in Clade V, instead of clustering with Panicoideae, Chloridoideae *FD* genes formed a sister group with Bambusoideae, indicating a complex evolutionary history within the Poaceae *FD* genes.

**Figure 2:**
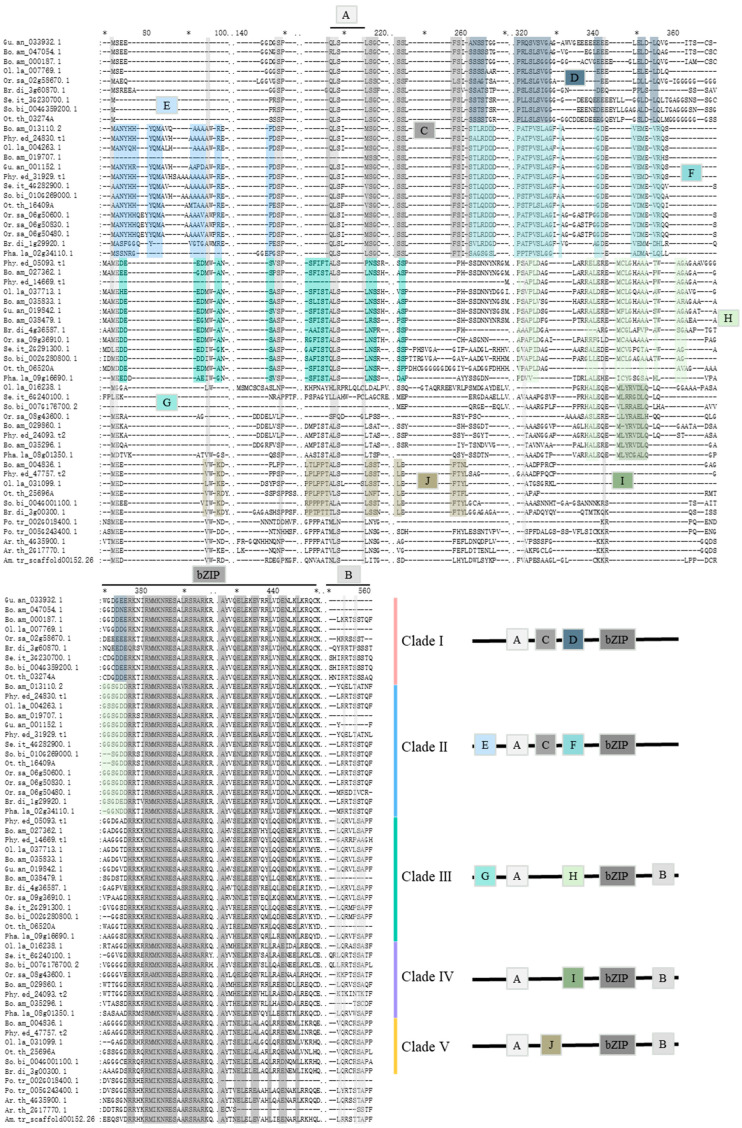
Protein sequence alignment and arrangements of amino acid motifs of the FD family members in bamboo and its related species. Different colors are conserved amino acid sequences in different clades. The conserved structural domains are in the boxes of same colors. The capital letters A to J denote distinct motifs, with their respective sequences displayed in Table S2. * was used to mark the positions of the amino acid sequences, with one * placed every 10 amino acids.

**Figure 3:**
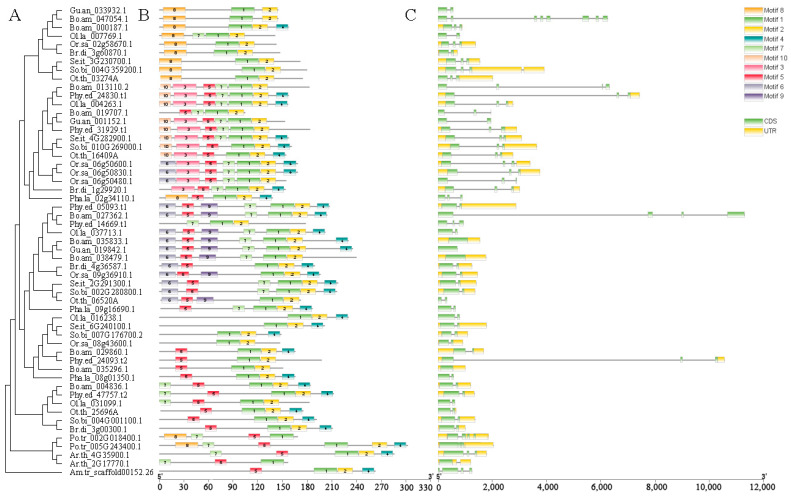
Conserved motifs and gene structures of the FD family members in bamboo and their related species. (**A**) A cladogram illustrating the phylogenetic relationship of FD protein of bamboo and its related species. (**B**) The distribution of conserved motifs of FD family members in bamboo and their related species. (**C**) The gene structures of FD family members in bamboo and their related species. The boxes and lines denote exons and introns, respectively. The scale on the bottom is in base pair (bp). The amino acid sequences of motifs 1 to 10 can be found in detail in Figure S3.

To explore the conserved motifs in the FD proteins in Poaceae, we performed sequence searches against the SMART and Pfam databases and carried out an MEME analysis (Figure 2, Figure 3 and Figure S2; Table S2). A total of ten conserved structural domains were identified. The comparison of domain arrangement across the five clades revealed a remarkable degree of structural domain conservation among the FD homologous proteins. Specifically, 33 amino acids (M_72_P_159_L_207_S_208_L_215_R_376_R_377_R_380_M_381_M_382_K_383_N_384_R_385_E_386_S_387_A_388_R_390_S_391_R_392_A_393_R_394_K_395_A_426_Y_427_E_430_L_431_E_432_E_434_V_435_L_438_E_441_N_442_L_445_), most of which belong to Motif A and the bZIP domain, were almost universally conserved across all sequences (Figure 2 and Figure 3). This observation supports the notion of a highly conserved structural configuration in FD homologous proteins.

Besides motifs shared by all clades, some motifs are shared by a subset of the five clades (Figure 2)—Clade I and Clade II share Motif C, and Clades III to V share Motif B. Other motifs were distributed consistently within certain clades: Motif D is present in only Clade I; Motifs E and F are present in Clade II; Motifs G and H are present in Clade III; Motif I is present in Clade IV; and Motif J is present in Clade V. This highlights the functional diversity of *FD* genes within different clades, with the consistent distribution of protein-conserved motifs implying close homology and similar gene functions within the same clade.

To gain further insights into the evolution of *FD* genes, we conducted an examination of the exon structure for each gene and, subsequently, mapped this information onto a phylogenetic tree. Our findings revealed distinct patterns of conservation and differentiation among genes belonging to different clades (Figure 3 and Figure S3). Within the Poaceae family, *FD* genes exhibited varying numbers of exons, ranging from one to eight. Notably, Clade V displayed the highest degree of gene structure conservation, as all genes in this clade contained two exons. Clade I and Clade II also exhibited relatively conserved gene structures, with most genes containing three exons, except for *Bo.am_047054.1*, which contained eight exons. Meanwhile, Clade III and Clade IV demonstrated a relatively consistent gene structure, with the majority of genes having two exons.

### 2.3. Evolutionary Pattern of FD-like Genes in Bamboos

**Figure 4:**
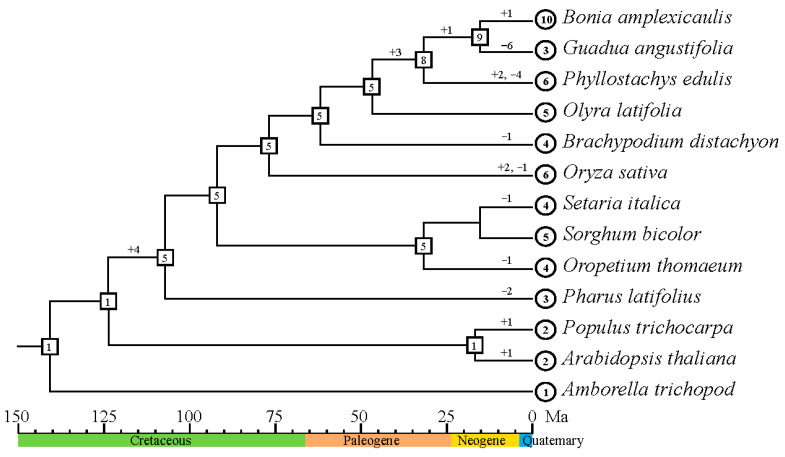
Copy number change in the *FD*-like gene family through evolution. Numbers in circles and rectangles represent the numbers of genes in extant and ancestral species, respectively. The plus (+) and minus (–) signs indicate the numbers of genes gained and lost since speciation.

To investigate the dynamic changes in the copy number of *FD* homologous genes during plant evolution, we conducted a comprehensive analysis using gene trees and species trees. Our findings unveiled intriguing patterns of gene duplication and loss events that have shaped the diversity of *FD* gene copies in different plant lineages (Figure 4). As stated before, one single copy of *FD* gene was present in the most recent common ancestor of all angiosperms. However, with the diversification of angiosperms, the number of *FD* gene copies increased to five in the most recent common ancestor of the Poaceae family. These results suggest that variations in *FD* gene copy numbers among extant plant species predominantly result from independent gene gains and losses throughout evolutionary history. Further exploration of copy number variations across Poaceae subfamilies revealed interesting differences. In subfamilies such as Pharoideae, Chloridoideae, Panicoideae, and Oryzoideae, the number of *FD* homologous genes remained constant or nearly constant compared to the inferred ancestral state, indicating a high level of conservation. In contrast, the *FD* homologous genes in Bambusoideae exhibited more significant fluctuations in copy numbers. The most recent common ancestor of the Bambusoideae possessed five copies of the *FD* gene, and during the past 45 million years, woody bamboos (*Phyllostachys edulis*, *Bonia amplexicaulis*, *Guadua angustifolia*) underwent drastic changes in copy numbers. In contrast, herbaceous bamboos (*Olyra latifolia*) did not experience significant changes in *FD* gene copy numbers during this period.

**Figure 5:**
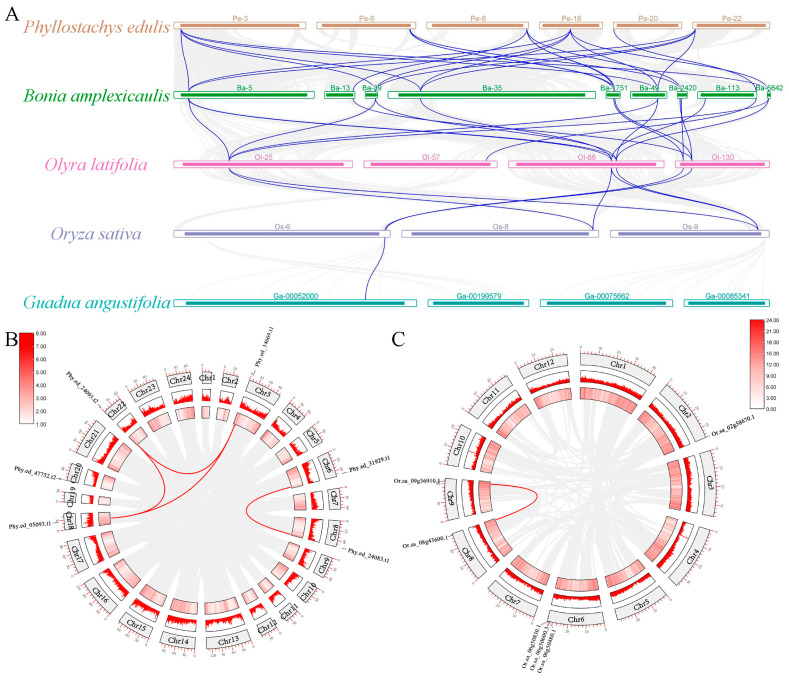
Analysis of chromosome distribution and intra-syntenic relationship of *FD* family in four bamboo species and rice. (**A**) Interlinear analysis of *P. edulis* (Pe), *Bonia amplexicaulis* (Ba), *Olyra latifolia* (Ol), *Oryza sativa* (Or), and *Guadua angustifolia* (Ga). (**B**) The chromosomal distribution of the *FD* gene in *P. edulis.* Gray lines indicate collinear relationship of all the members of *P. edulis*, red lines represent collinear relationship between the members of *FD* family, and red areas show the gene density. (**C**) The chromosomal distribution of the *FD* gene in rice. Gray lines indicate collinear relationship of all the members of rice, red lines represent collinear relationship between the members of *FD* family, and red areas show the gene density.

To investigate the expansion pattern of the *FD* gene in the Bambusoideae, we conducted inter-species synteny analysis to identify the types of gene duplication events between the bamboo subfamily and rice. Our analysis identified three pairs of homologous genes shared between the bamboo subfamily and the rice genome (Figure 5A), denoted as *Phy.ed_31929.t1-Bo.am_013110.2*/*Bo.am_019707.1-Ol.la_004263.1*, *Phy.ed_14669.t1*/*Phy.ed_05093.t1*/*Phy.ed_24093.t2*-*Bo.am_027362.1/Bo.am_035833.1/Bo.am_038479.1/Bo.am_029860.1/Bo.am_035296.1-Ol.la_037713.1/Ol.la_016238.1-Or.sa_09g36910.1/Or.sa_08g43600.1*, and *Phy.ed_47757.t2-Bo.am_004836.1-Ol.la_031099.1*.

Within the *FD* gene family in *P. edulis*, we observed three gene duplication events. Combined with rice gene duplicate, these events were identified as follows: one was a Poaceae-wide gene duplication event leading to the formation of Clade III and Clade IV, and two were Bambusoideae gene duplication events resulting in the generation of *Phy.ed_05093.t1/Phy.ed_14669.t1* and *Phy.ed_31929.t1/Phy.ed_24830.t1* (Figure 5B,C). Remarkably, all these duplication events occurred between different chromosomes. Our analyses revealed that segmental duplication serves as the primary mechanism driving the duplication of *FD* genes within the Bambusoideae in a relatively short period of time. This mechanism has facilitated the expansion of *FD* gene copies, contributing to the diversification and functional differentiation within the *FD* gene family in Bambusoideae.

To assess the environmental selection pressure acting on *FD* homologous genes after duplication, we conducted a selective constraint analysis by calculating the rates of synonymous substitutions (Ks) and non-synonymous substitutions (Ka), as well as the ratio of these two values (Ka/Ks) for each clade (Table 1). All branches investigated in this study have Ka/Ks ratios less than one, suggesting that purifying selection has been acting on these branches to retain functional stability and preserve the essential roles of *FD* genes. Notably, Clade II exhibited a significantly smaller Ka/Ks ratio compared to the other clades, suggesting that it experienced particularly strong negative selection forces (Table 1). This implies that Clade II genes have undergone stringent selective pressures to maintain their critical functions and conserve their protein sequences across evolutionary time.

### 2.4. Identification of Cis-Elements of FD-like Genes

**Figure 6:**
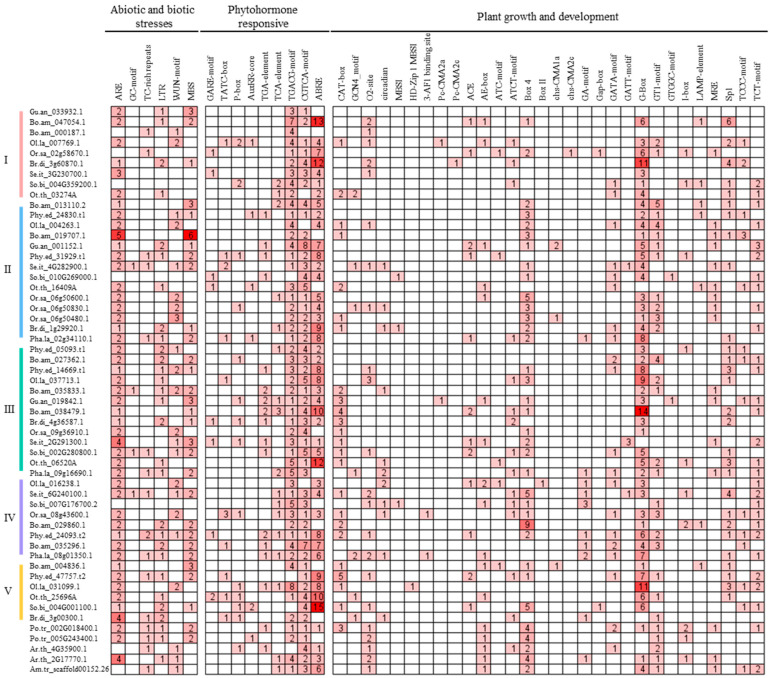
*Cis*-regulatory element analysis of *PhyFD* gene promoters. Counts in the red boxes are the number of *cis*-elements. Darker red means more promoters.

To explore the potential regulatory mechanism of *FD* genes, the promoter regions (2000 bp upstream sequence from the start codon of the genomic DNA sequence) of the *FD* genes were submitted to the PlantCARE database. *Cis*-elements involved in abiotic and biotic stress responses, phytohormone responses, and plant growth and development were identified (Figure 6). Among the abiotic and biotic stress response elements, anaerobic inducible elements (ARE and GC-motif), defense and stress response elements (TC-rich repeats), low-temperature responsive (LTR) elements, wound-responsive elements (WUN-motif), and drought-inducible elements (MBS) were found in 44, 16, 29, 22, and 30 *FD* genes, respectively. Meanwhile, gibberellin response elements (GARE-motif, TATC-box, and P-box), auxin response elements (AuxRR-core and TGA-element), salicylic acid response elements (TCA-element), and MeJA response elements (TGACG-motif and CGTCA-motif) were detected in 24, 22, 17, and 51 *FD* genes, respectively. In addition, light response elements were detected in the *FD* gene promoters, with the largest number and the widest range of G-box elements. Other plant growth and development-related elements, such as meristem expression elements (CAT-box and GCN4_motif), zinc metabolism elements (O2-site), circadian control elements (circadian), flavonoid biosynthetic gene regulation elements (MBSI), and palisade mesophyll cell differentiation elements (HD-Zip 1), were observed in 24, 22, 13, 3, and 1 *FD* gene promoters, respectively. These elements indicate that *FD* genes may be involved in plant growth and development, hormone and defense signaling, and adaptation to environmental changes.

### 2.5. Expression Pattern of FD-like Genes

Expression patterns of *FD* genes were examined across different plant organs in *Arabidopsis*, poplar, rice, and moso bamboo (Figure 1). In general, *FD* genes are expressed in both vegetative and reproductive organs. In *Arabidopsis*, both *FD* genes exhibit higher expression levels in shoot apical meristems and inflorescences, while in poplar and rice, higher *FD* expression is observed in roots. In bamboo, *Phy.ed_31929.t1*, *Phy.ed_05093.t1*, *Phy.ed_14669.t1*, and *Phy.ed_24093.t2* are expressed in both flowering and non-flowering organs, with higher expression levels in flowering leaves. Meanwhile, the expression levels of *Phy.ed_24830.t1* and *Phy.ed_47757.t2* are extremely low; thus, those two genes were not selected in downstream analyses.

**Figure 7:**
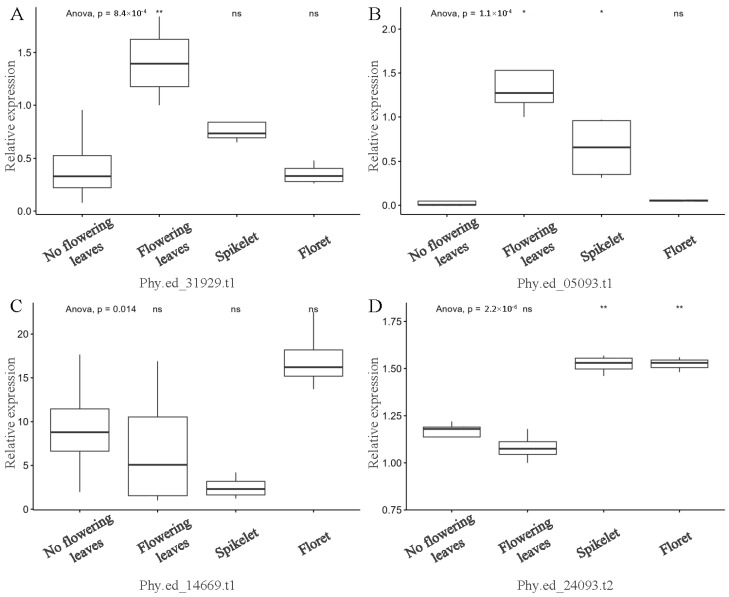
qRT-PCR expression analysis of selected *FD* genes in various organs before and during flowering. (**A**) Relative expression levels of *Phy.ed_31929.t1*. (**B**) Relative expression levels of *Phy.ed_05093.t1*. (**C**) Relative expression levels of *Phy.ed_14669.t1*. (**D**) Relative expression levels of *Phy.ed_24093.t2*. * means the *p*-value < 0.05 in the anova (various organs during flowering vs. leaves during the non-flowering period), ** means the *p*-value < 0.01, and ns means no significant difference.

In our subsequent investigations, we delved deeper into the expression patterns of four *FD* genes in moso bamboo across various tissues, including leaves in the non-flowering period, as well as leaves and other reproductive organs in the flowering period. We employed qRT-PCR to validate and complement the transcription results obtained from public RNA-seq data. The qRT-PCR findings were largely consistent with the initial transcriptional analysis (Figure 7). Specifically, *Phy.ed_31929.t1* exhibited significantly higher expression levels in the flowering leaves. *Phy.ed_05093.t1* displayed the same pattern, with lower expression levels observed in the non-flowering leaves. *Phy.ed_14669.t1* and *Phy.ed_24093.t2* were found to be expressed in all organs, resembling the expression patterns observed in the other three species (*Arabidopsis*, poplar, and rice).

### 2.6. Ectopic Expression of PhyFD in Arabidopsis

**Figure 8:**
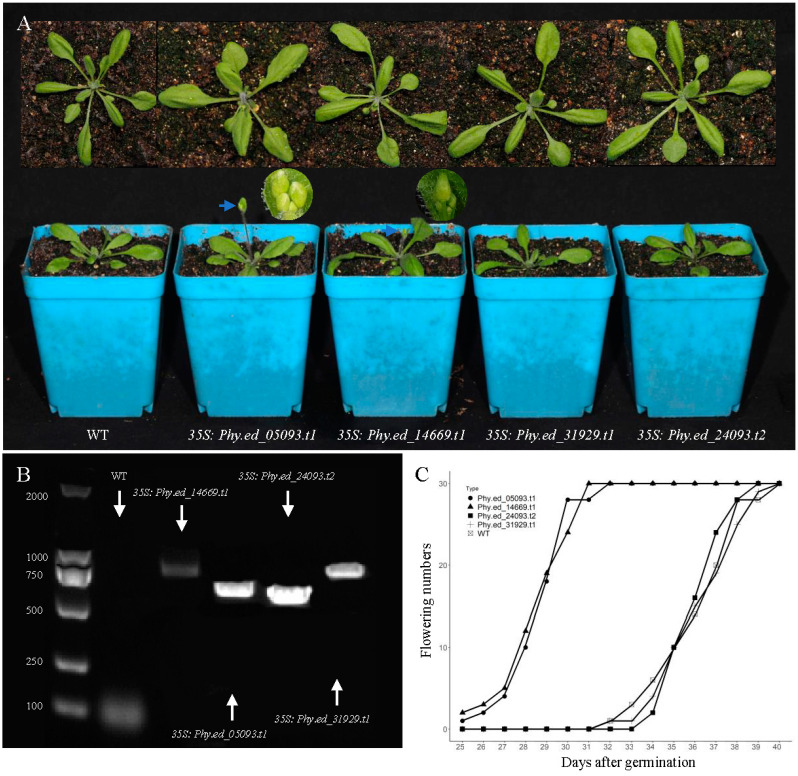
Analysis of an early flowering phenotype by overexpression of *FD* in *Arabidopsis*. (**A**) Phenotype of overexpression of target genes (30 days after seedling). (**B**) Results of PCR verification experiment. (**C**) The number of flowers during 25–40 day seedling of the overexpression lines in *Arabidopsis* (with 30 replicates per overexpression line).

To further investigate the function of the *PhyFD* genes in bamboo, *Arabidopsis* overexpression lines were created, each transformed with one of the four *PhyFD* genes driven by a 35S promoter. Flowering phenotypic traits in the overexpression and control lines were observed and recorded under long-day conditions (Figure 8). Compared to the wild-type plants, the overexpression of *Phy.ed_05093.t1* and *Phy.ed_14669.t1* significantly promotes flowering in *Arabidopsis*. Specifically, flower developments were observed as early as 25 days after sowing in these two overexpression lines, whereas the first flower did not appear in the wild-type *Arabidopsis* line until 32 days after sowing. However, no significant changes in flowering time were observed in other *Arabidopsis 35S:PhyFD* lines.

## 3. Discussion

### 3.1. Evolutionary History of the FD-like Genes in Bambusoideae

The *bZIP* superfamily plays a critical role in the growth and development of plants, exhibiting distinctive expansion and evolution in angiosperms [14]. Our exploration of *FD*-like genes in Bambusoideae necessitates an initial focus on the *bZIP* gene family. In this study, we conducted a comprehensive analysis to identify *FD* homologous genes in four bamboo genomes, resulting in the identification of 24 genes distributed across *Bonia amplexicaulis* (10 genes), *Guadua angustifolia* (3 genes), *Olyra latifolia* (5 genes), and *Phyllostachys edulis* (6 genes). These genes were further categorized into five clades based on their sequence similarities and phylogenetic relationships.

Throughout the evolutionary history of Poaceae, *FD* genes experienced four duplication events and multiple independent loss events, leading to the current gene content landscape across diverse Poaceae lineages. Clade V, which is the sister clade to all the others, lacks genes from three investigated species, i.e., *G. angustifolia*, rice, and *P. latifolius.* A Poaceae-wide duplication event, which happened after the divergence of Clade V and the ancestor of all the other clades, created the ancestral genes of two large monophyletic groups (Clade I + Clade II and Clade III + Clade VI). A subsequent duplication event created the rest of the four clades (Clades I to IV) that are present in the current Poaceae species. Notably, basal Poaceae lineages such as *Oropetium thomaeum* and *Pharus latifolius* lack genes from Clade I, and *O. thomaeum* also lacks members in Clade IV. This analysis led us to hypothesize that all *FD* genes share a common ancestor, with the ancestral protein sequence likely to contain the bZIP, Motif A, and Motif B structural domains.

Additionally, our findings revealed intriguing dynamics between the bamboo subfamily and other subfamilies in Poaceae. While the subfamilies outside of bamboo maintained a relatively stable copy number of *FD* genes, the bamboo subfamily underwent rapid evolution, resulting in significant changes in gene numbers. For instance, in *B. amplexicaulis*, the number of *FD* genes increased from 5 to 10 within a relatively short time frame of approximately 50 million years. In moso bamboo, two fragment duplications were predicted within the past 25 million years followed by four independent loss events, contributing to the formation of the six existing copies of *FD* genes in moso bamboo. This remarkable expansion can be attributed to the woody bamboo ancestor undergoing three independent heterologous polyploidization events, resulting in the formation of the hexaploid woody bamboo. Presumably, these polyploidization events played a crucial role in driving the rapid evolution and diversification of *FD* genes within the bamboo subfamily, possibly in response to the Oligocene climate change [17].

### 3.2. Relationship Between the Organization of Structural Domains and Gene Functional Diversification

Our intron–exon structure analyses unveiled that the single ancestral *FD* gene in the MRCA of Poaceae was encoded by two exons. However, within Bambusoideae, numerous gene duplication events introduced introns into different positions within the exons of the ancestral gene. This process led to the generation of structural diversity among *FD* genes within Bambusoideae. Additionally, the ancestral gene was predicted to contain Motif B, however, this motif was lost in the ancestor of Clades I and II, while a new motif, Motif C, was formed. Like Clades I and II, conserved motifs formed within each individual clade. The formation and deletion of these gene structures may have resulted in functional diversity of the *FD* homologous genes. For instance, in rice, *Or.sa_09g36910.1* in Clade III has the ability to activate the expression of *AP1/FUL* genes, thereby promoting rice flowering [5]. Conversely, the function of *Or.sa_06g50600.1* in Clade III is associated with leaf development, showcasing the diverse functional roles of *FD* homologous genes resulting from the intricate interplay of motif conservation and alterations.

To evaluate the environmental selection pressure on duplicated *FD* homologous genes, we conducted a selective constraint analysis by computing the rates of synonymous substitutions (Ks) and non-synonymous substitutions (Ka), along with their ratio (Ka/Ks) for each clade (Table 1). A Ka/Ks ratio greater than one indicates a positive selection effect, a ratio of one signifies a neutral selection effect, and a ratio less than one suggests a negative selection effect, namely purifying selection [18]. All branches examined in this study exhibit Ka/Ks ratios below one, indicating that purifying selection has been active in preserving functional stability and upholding the essential functions of *FD* genes.

### 3.3. The Divergence of FD-like Gene Expression Pattern

The function of the *FD* gene family is associated with plant flowering, with its expression levels closely correlated to its function. Gene expression primarily relies on *cis*-regulatory elements within the promoter region, prompting us to predict *cis*-regulatory elements in the *PhyFD* gene’s promoter and conduct spatiotemporal expression analysis. Flowering and dormancy are of significant biological importance for the reproduction and survival of woody plants, with hormones playing a crucial role as internal factors [3]. Gibberellic acid (GA), a key hormone in flowering regulation, works in antagonism with abscisic acid (ABA), jointly controlling plant flowering [19]. The *PhyFD* gene’s promoter contains a significant number of hormone-responsive elements, suggesting its involvement in hormone pathways. Specifically, the six *PhyFD* genes identified in this study house over 22 ABA-responsive elements, which may participate in ABA-mediated growth processes, such as bud dormancy.

Recent results revealed that the *FD* homologous genes in *Arabidopsis*, poplar, and rice exhibit expression in all tissues [14,16,20]. In *Arabidopsis* and rice, higher expression levels are detected in shoot apical meristems and inflorescences, while in poplar, *FD* expression is higher in the roots. In bamboo, *Phy.ed_31929.t1* attains peak expression in the inflorescence, while *Phy.ed_05093.t1* and *Phy.ed_14669.t1* show the highest expression levels in the leaves about to flower and in the flowering leaves, respectively. Distinct spatiotemporal expression among *PhyFD* genes indicates that subfunctionalization or neofunctionalization events may have occurred among these copies.

Furthermore, real-time fluorescence quantitative PCR results demonstrated that the expression pattern of the *FD* gene in *P. edulis* mirrors that of *Arabidopsis*, poplar, and rice. This alignment of expression patterns reinforced the notion of functional conservation and divergence of *FD* genes across these plant species. These results further emphasize the diverse and specialized roles of *FD* genes in *P. edulis*, especially during the flowering stage. The distinct expression patterns in different organs of flowering and non-flowering plants highlight the potential regulatory functions of *FD* genes in the developmental processes associated with flowering.

### 3.4. Functional Analysis of PhyFD Genes

To further investigate the role of *FD* in the transcriptional regulation of flowering time, *FD* was overexpressed in *Arabidopsis* (WT). Overexpression of *Phy.ed_05093.t1* and *Phy.ed_14669.t1* promotes early flowering in *Arabidopsis* by 5–7 days, while overexpression of *Phy.ed_31929.t1* and *Phy.ed_24093.t2* shows no significant phenotypic changes, indicating that they may play distinct regulatory roles in bamboo development. While it is currently challenging to uncover the intrinsic molecular mechanisms underlying bamboo flowering, the research conducted in this experiment provides initial insights into the roles of the *Phy.ed_05093.t1* and *Phy.ed_14669.t1* genes in bamboo flowering. This sets a foundation for further studies.

## 4. Materials and Methods

### 4.1. Identification of Members of the bZIP Gene Family

From the Plant Transcription Factor Database v5.0 (http://planttfdb.gao-lab.org/family.php?fam=bZIP) (accessed on 19 March 2023) [21], bZIP protein sequences were obtained from the following 9 species: the basal angiosperms *Amborella trichopod*, 2 dicotyledons including *Arabidopsis thaliana* and *Populus trichocarpa*, and 6 monocotyledons including *Brachypodium distachydium*, *Oropetium thomaeum*, *Oryza sativa*, *Pharus latifolius*, *Setaria italica*, and *Sorghum bicolor*. The complete gene data of three woody bamboos (*Phyllostachys edulis*, *Bonia amplexicaulis*, *Guadua angustifolia*) and one herbaceous bamboo (*Olyra latifolia*) were downloaded from GIGADB [22] and Bamboo genome database [23]. In this study, the protein sequences from each species were used as the database, with the *Arabidopsis* FD protein serving as the input sequence. Similarity sequences of bZIP proteins were obtained by native blastp with e-value < 1 × 10^−5^ as a threshold, and the resulting dataset was screened to remove sequences without bZIP domains using the programs on the HMMER (version 3.3.2) [24] and CDD [25] websites. Finally, a total of 1333 bZIP proteins were identified for subsequent analysis, including 37 copies in *Amborella trichopod*, 74 in *Arabidopsis thaliana*, 92 in *Populus trichocarpa*, 90 in *Brachypodium distachydium*, 73 in *Oropetium thomaeum*, 95 in *Oryza sativa*, 84 in *Pharus latifolius*, 92 in *Setaria italica*, 96 in *Sorghum bicolor*, 179 in *Phyllostachys edulis*, 210 in *Bonia amplexicaulis*, 114 in *Guadua angustifolia*, and 97 *in Olyra latifolia*.

### 4.2. Sequence Alignment and Phylogenetic Analysis

The MAFFT (version 7.487) algorithm was used to perform multiple sequence alignment of all 1333 proteins and to generate a full-length matrix [26]. To obtain a matrix containing conserved bZIP domains, the matrix was processed using trimAl (v1.2rev57) with the following parameters: -cons 10.0 -gt 0.2 -st 0.0 -w 1.0.

The bZIP phylogenetic tree of 13 species was constructed using the maximum likelihood (ML) method implemented in RAxML (version 7.0.4) with JTT, the best model estimated by ModelFinder (version 2.2.0) [27]. For the phylogenetic analysis of FD, the full-length protein sequence alignment matrix was used as the input for two phylogenetic inference algorithms—a ML tree constructed with the maximum likelihood method and a Bayesian tree constructed using Bayeux Inference method (BI). The ML tree was constructed using RAxML [28] with JTT+I+G4, the best model estimated by ModelFinder [27]. BI analysis was performed with MrBayes version 3.2.6, starting with random trees and sampling a tree every 1000 generations for 5,000,000 generations.

### 4.3. Conserved Domain and Gene Structure Analysis

Sequence alignment of all FD proteins was conducted using MAFFT (version 7.487) with default parameters. The alignment results were further refined and manually improved using GeneDoc (version 2.6.0.2). To predict and analyze the conserved structure of amino acids encoded by *FD* homologous genes, we utilized the online software MEME (version 5.0.5) [29] (available at https://meme-suite.org/meme/tools/meme) (accessed on 1 March 2023) with -protein -oc. -nostatus -time 14400 -mod zoops -nmotifs 8 -minw 6 -maxw 50 -objfun classic -markov_order 0.

### 4.4. Expression Profile Analysis

In this study, we investigated developmental stages of moso bamboo ranging from seed and seedling to above-ground tissues such as leaves, stems, and flowering. To gain further understanding of gene expression dynamics during these developmental stages, we employed the ePlant 2.0 [30] tool to provide detailed visualizations and annotations. The raw RNA-seq data for moso bamboo were obtained from the NCBI Sequence Read Archive (SRA) under the accession number GSE121216 [31]. To quantify the expression levels of transcripts, we utilized Hisat2 (version 2.1.0) [32] software for read alignment to the reference genome. Subsequently, the Relative Per Kilobase of transcript per Million mapped reads (RPKM) values were calculated to quantify the expression levels of transcripts. RPKM is a normalized measure of gene expression that considers both the gene length and the total number of reads mapped to the gene, allowing for a reliable comparison of expression levels across different genes.

### 4.5. Gene Duplication and Selection Pressure

First, we used blastp to search the whole genome protein sequence of *P. edulis*, the E value was 1 × 10^−5^, and we identified >50%. Then, collinear regions were detected using the default parameters of MCScanX (version 1.1.11) [33]. Finally, TBtools [34,35] was used to create collinearity analysis plots based on information on collinear pairs and genetic positions.

Selection pressure and occurrence of duplication events were estimated on the dataset by calculating non-synonymous substitution (Ka) and synonymous (Ks) rates between collinear pairs using KaKs_Calculator 2.0 [36].

### 4.6. Plant Materials

*Arabidopsis* plants were grown under long daylight exposure (16 h light/8 h dark) in light growth incubator maintained at 23 °C with 40 to 50% humidity and an irradiance of approximately 118 mmol·m^−2^·s^−1^. In addition, the flower buds and flowers of *P. edulis* at different flowering developmental stages were collected in Dajing County, Guilin (E 110°170′–110°470′; N 25°04′–25°48′) in Guangxi Zhuang Autonomous Region from April to August 2021.

### 4.7. Real-Time Fluorescent Quantitative PCR

According to previous methods, the total RNA of bamboo tissues was extracted with the Plant total RNA extraction kit (ER501-01; TRANS, Beijing, China). RNA integrity was assessed using the RNA Nano 6000 Assay Kit of the Bioanalyzer 2100 system (Agilent Technologies, Palo Alto, CA, USA), and the quality was detected by 1% agarose gel electrophoresis. cDNAs were then synthesized with EasyScript^®^ All-in-One First-Strand cDNA Synthesis SuperMix for qPCR (One-Step gDNA Removal) HiScript QRT SuperMix for qPCR (+gDNA wiper) (AE341; TRANS, Beijing, China). The primer design was conducted using Primer Premier 5 software, with the product length restricted to 100–300 base pairs. The *PheTFL1* gene of moso bamboo was used as an internal reference gene [37]. The primers utilized in this experiment were synthesized by Beijing Qingke Biotechnology Co., Ltd. (Beijing, China).

According to the gene-specific primers (Table S3), the expression of *FD* family genes in bamboo was conducted using the CFX96 automatic fluorescence quantitative PCR instrument (Bio-Rad, Hercules, CA, USA) with PerfectStart^®^ Green qPCR SuperMix (AQ601; TRANS, Beijing, China). Triplicate RT-PCR of each sample was performed. The reaction volume was 20 μL and included 10 μL of 2× PerfectStart Green qPCR SuperMix, 8.2 μL of ddH_2_O, 1 μL of cDNA template, and 0.4 μL of forward and reverse primers. The total reaction conditions included a pre-denaturation step at 95 °C for 2 min and 40 cycles of RT-PCR amplification (denaturation at 95 °C for 5 s and annealing at 60 °C for 34 s). Finally, the 2^−ΔΔCt^ method was used for analysis [38]. Three biological and three technological repeats were performed in RT-PCR.

### 4.8. Overexpression Experiments

Leaf cDNA from *P. edulis* was used as template to amplify sequences of targeted *PhyFD* genes using PCR with specific primers (Table S4). The product was initially cloned into pGEM-T Easy vector. It was then ligated with the *35S:pCAMBIA1301* vector using the restriction endonuclease Bam HI. The *35S:PhePhyFD* construct was introduced into wild-type *Arabidopsis* plants (Columbia-0) through an *Agrobacterium*-mediated floral dip transformation method [39]. After PCR detection, positive transgenic strains were screened out (Figure 8).

## 5. Conclusions

In this study, we identified 24 *FD* homologous genes across four bamboo species, which were subsequently grouped into five clades. Our analysis revealed that these *FD* homologous genes were independently lost multiple times during the evolutionary history of the Poaceae family. Through intron–exon structure analysis, we hypothesized that the *FD* homologous genes in Poaceae share a common evolutionary origin. The promoters of these genes contained numerous hormone-responsive elements, suggesting their potential involvement in hormone signaling pathways. Overexpression of *Phy.ed_05093.t1* and *Phy.ed_14669.t1* resulted in a 5–7 day advancement in flowering time in *Arabidopsis*, while overexpression of *Phy.ed_31929.t1* and *Phy.ed_24093.t2* did not lead to significant phenotypic changes, indicating that these genes may play distinct regulatory roles in bamboo development.

## Figures and Tables

**Table 1:** Estimated selection pressure (Ka/Ks) of direct/collateral homologous *FD* gene pairs in Poaceae plants.

Clade	Seq_1	Seq_2	Ka	Ks	Ka/Ks
Clade II	Phy.ed_31929.t1	Bo.am_013110.2	0.067058	0.176543	0.379837
	Phy.ed_31929.t1	Bo.am_019707.1	0.032379	0.154199	0.209982
	Phy.ed_31929.t1	Ol.la_004263.1	0.166616	0.371712	0.448239
	Bo.am_013110.2	Ol.la_004263.1	0.144827	0.311292	0.465244
	Bo.am_019707.1	Ol.la_004263.1	0.074487	0.179288	0.415459
Clade III	Phy.ed_14669.t1	Bo.am_027362.1	0.291472	0.600556	0.485337
	Phy.ed_14669.t1	Bo.am_035833.1	0.194275	0.265512	0.731697
	Phy.ed_14669.t1	Bo.am_038479.1	0.192163	0.276153	0.695857
	Phy.ed_14669.t1	Ol.la_037713.1	0.138899	0.265594	0.522975
	Phy.ed_14669.t1	Or.sa_09g36910.1	0.312352	0.397803	0.785192
	Bo.am_027362.1	Ol.la_037713.1	0.16103	0.23471	0.686082
	Bo.am_035833.1	Ol.la_037713.1	0.097434	0.204982	0.47533
	Bo.am_038479.1	Ol.la_037713.1	0.176587	0.326993	0.540032
	Bo.am_027362.1	Or.sa_09g36910.1	0.292893	0.421393	0.695059
	Bo.am_035833.1	Or.sa_09g36910.1	0.205465	0.379931	0.540796
	Bo.am_038479.1	Or.sa_09g36910.1	0.273412	0.481567	0.567755
	Ol.la_037713.1	Or.sa_09g36910.1	0.223141	0.367388	0.607372
	Phy.ed_05093.t1	Bo.am_027362.1	0.126013	0.146983	0.85733
	Phy.ed_05093.t1	Bo.am_035833.1	0.0732	0.100029	0.731787
	Phy.ed_05093.t1	Bo.am_038479.1	0.150125	0.228915	0.655813
	Phy.ed_05093.t1	Ol.la_037713.1	0.094446	0.193782	0.48738
	Phy.ed_05093.t1	Or.sa_09g36910.1	0.259631	0.359129	0.722947
Clade IV	Phy.ed_24093.t2	Bo.am_029860.1	0.178447	0.219873	0.81159
	Phy.ed_24093.t2	Bo.am_035296.1	0.14802	0.21697	0.682211
	Phy.ed_24093.t2	Ol.la_016238.1	0.219559	0.366693	0.598754
	Phy.ed_24093.t2	Or.sa_08g43600.1	0.454448	0.715968	0.634732
	Bo.am_029860.1	Ol.la_016238.1	0.128375	0.208648	0.615269
	Bo.am_035296.1	Ol.la_016238.1	0.464204	0.678203	0.684462
	Bo.am_029860.1	Or.sa_08g43600.1	0.309042	0.51619	0.598698
	Bo.am_035296.1	Or.sa_08g43600.1	0.382542	0.638793	0.598852
	Ol.la_016238.1	Or.sa_08g43600.1	0.381029	0.520243	0.732405
Clade V	Phy.ed_47757.t2	Bo.am_004836.1	0.053177	0.092482	0.575001
	Phy.ed_47757.t2	Ol.la_031099.1	0.102647	0.199515	0.51448
	Bo.am_004836.1	Ol.la_031099.1	0.122386	0.193244	0.633326

## Data Availability

Data are contained within the article or Supplementary Materials.

## Supplementary Materials

The following supporting information can be downloaded at https://www.mdpi.com/article/10.3390/ijms252313062/s1.
