# Supplementary material for: Genome-Wide Identification and Expression Analysis of FD Gene Family in Bamboos
Source: Int J Mol Sci. 2024 Dec 5;25(23):13062. doi: 10.3390/ijms252313062 (PMC11641677; doi:10.3390/ijms252313062)
Supplement: Supplementary file 1 [file ijms-25-13062-s001.zip › Supplementary files-revision/Supplementary Figures-Genome‐wide identification and expression analysis of the FD gene family in bamboo.pptx]

## Slide 1
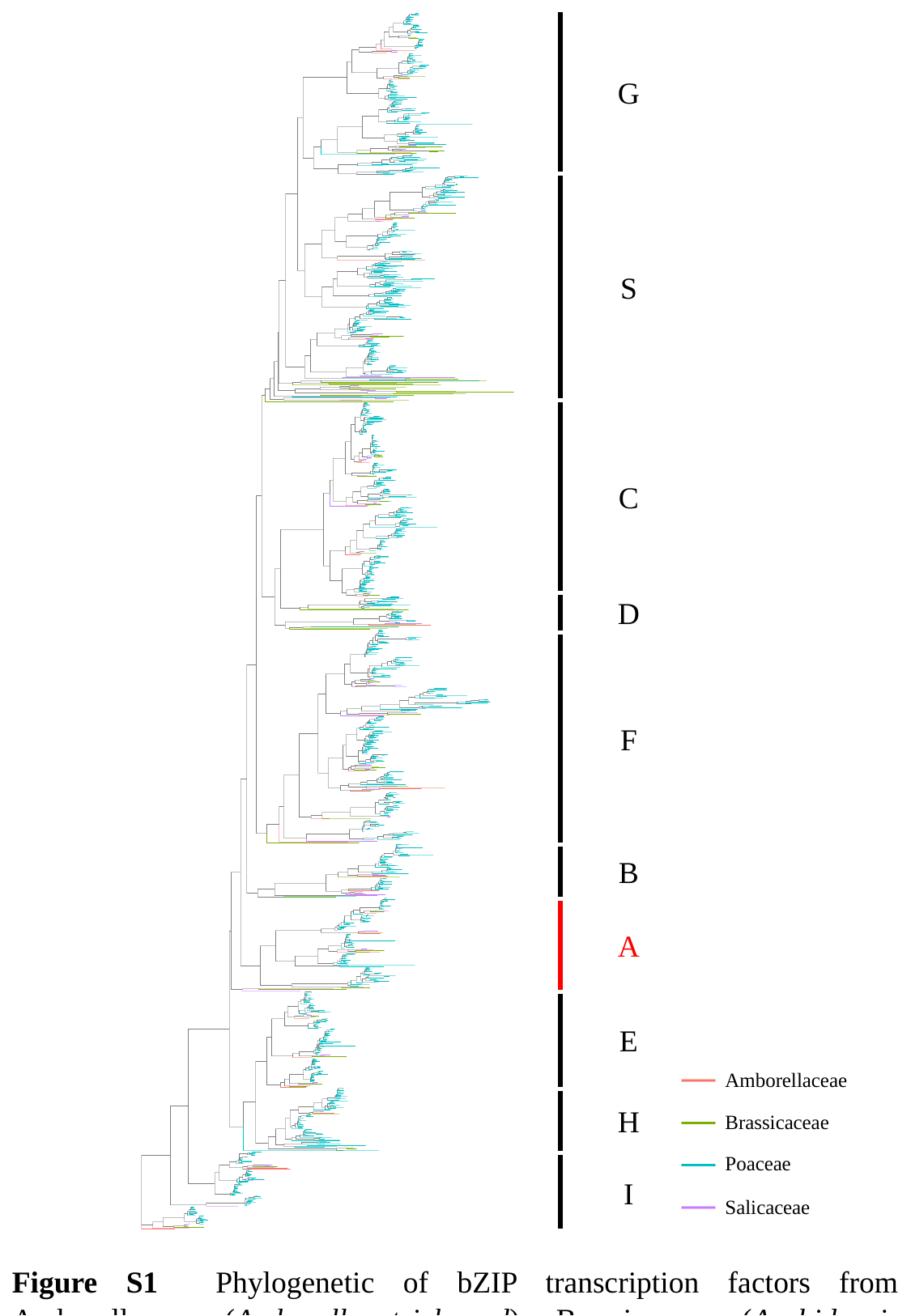

G
S
C
D
F
B
A
E
H
I
Amborellaceae
Brassicaceae
Poaceae
Salicaceae
Figure S1 Phylogenetic of bZIP transcription factors from Amborellaceae (Amborella trichopod), Brassicaceae (Arabidopsis thaliana), Salicaceae (Populus trichocarpa), Poaceae (Pharus latifolius, Oropetium thomaeum, Sorghum bicolor, Setaria italica, Oryza sativa, Brachypodium distachyon, Olyra latifolia, Phyllostachys edulis, Guadua_angustifolia, Bonia amplexicaulis). The predicted full-length amino acid sequences of and 37 AmTr bZIP 74 ArTh bZIP, 210 BoAm bZIP, 87 BrDi bZIP, 114 GuAn bZIP, 97 OlLa Bzip, 89 OrSa bZIP, 154 PhEd bZIP, 92 SeIt bZIP, and 96 SoBi bZIP were used to construct a phylogenetic tree using iqtree by the ML method.

## Slide 2
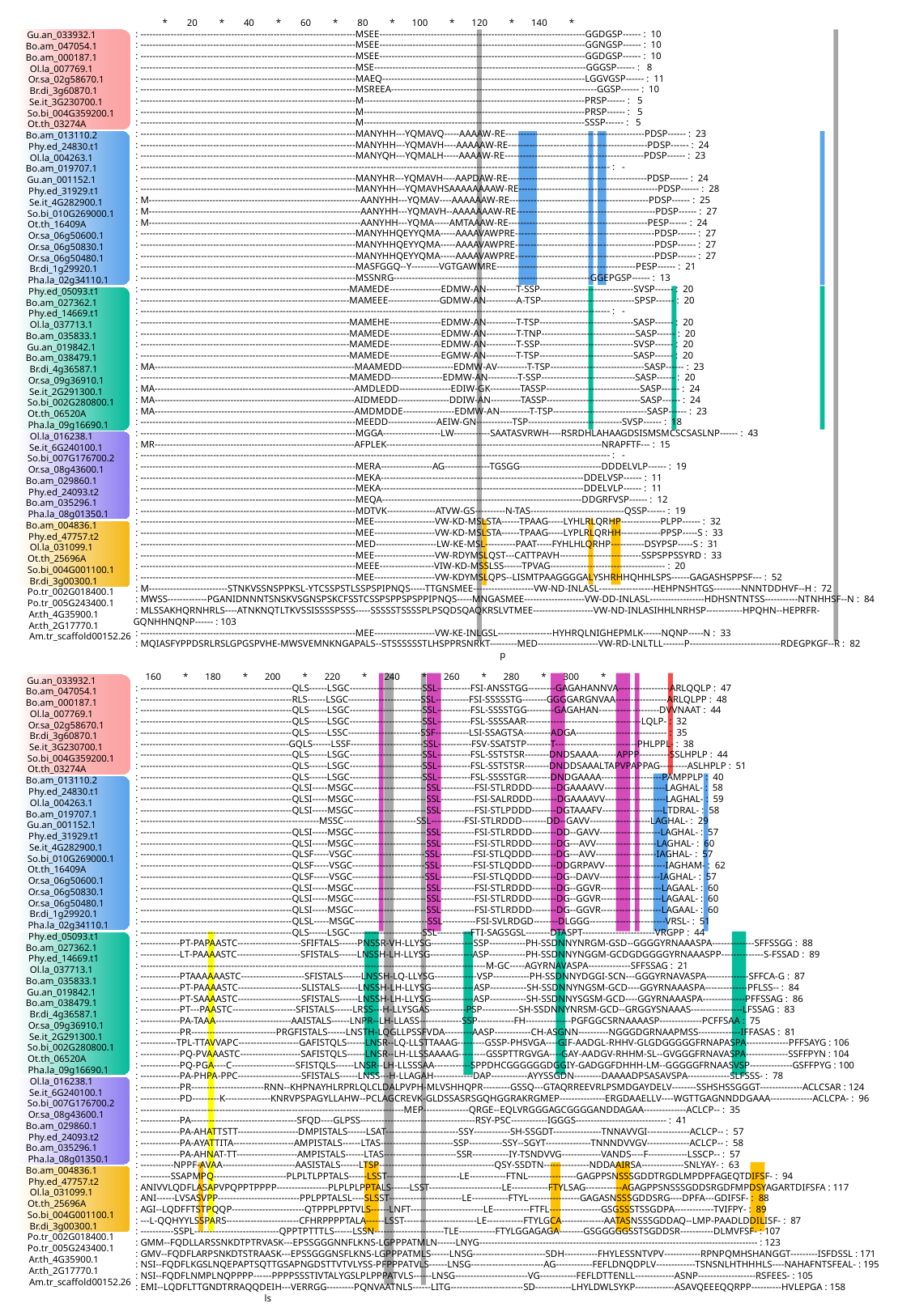

* 20 * 40 * 60 * 80 * 100 * 120 * 140 *
 : -----------------------------------------------------------------------MSEE--------------------------------------------------------------------GGDGSP------ : 10
 : -----------------------------------------------------------------------MSEE--------------------------------------------------------------------GGNGSP------ : 10
 : -----------------------------------------------------------------------MSEE--------------------------------------------------------------------GGDGSP------ : 10
 : -----------------------------------------------------------------------MSE----------------------------------------------------------------------GGGSP------ : 8
 : -----------------------------------------------------------------------MAEQ-------------------------------------------------------------------LGGVGSP------ : 11
 : -----------------------------------------------------------------------MSREEA--------------------------------------------------------------------GGSP------ : 10
 : -----------------------------------------------------------------------M-------------------------------------------------------------------------PRSP------ : 5
 : -----------------------------------------------------------------------M-------------------------------------------------------------------------PRSP------ : 5
 : -----------------------------------------------------------------------M-------------------------------------------------------------------------SSSP------ : 5
 : -----------------------------------------------------------------------MANYHH---YQMAVQ-----AAAAW-RE----------------------------------------------PDSP------ : 23
 : -----------------------------------------------------------------------MANYHH---YQMAVH----AAAAAW-RE----------------------------------------------PDSP------ : 24
 : -----------------------------------------------------------------------MANYQH---YQMALH-----AAAAW-RE----------------------------------------------PDSP------ : 23
 : ----------------------------------------------------------------------------------------------------------------------------------------------------------- : -
 : -----------------------------------------------------------------------MANYHR---YQMAVH----AAPDAW-RE----------------------------------------------PDSP------ : 24
 : -----------------------------------------------------------------------MANYHH---YQMAVHSAAAAAAAAW-RE----------------------------------------------PDSP------ : 28
 : M----------------------------------------------------------------------AANYHH---YQMAV----AAAAAAW-RE----------------------------------------------PDSP------ : 25
 : M----------------------------------------------------------------------AANYHH---YQMAVH--AAAAAAAW-RE----------------------------------------------PDSP------ : 27
 : M----------------------------------------------------------------------AANYHH---YQMA-----AMTAAAW-RE----------------------------------------------PESP------ : 24
 : -----------------------------------------------------------------------MANYHHQEYYQMA-----AAAAVAWPRE----------------------------------------------PDSP------ : 27
 : -----------------------------------------------------------------------MANYHHQEYYQMA-----AAAAVAWPRE----------------------------------------------PDSP------ : 27
 : -----------------------------------------------------------------------MANYHHQEYYQMA-----AAAAVAWPRE----------------------------------------------PDSP------ : 27
 : -----------------------------------------------------------------------MASFGGQ--Y---------VGTGAWMRE----------------------------------------------PESP------ : 21
 : -----------------------------------------------------------------------MSSNRG-----------------------------------------------------------------GGEPGSP------ : 13
 : ---------------------------------------------------------------------MAMEDE-----------------EDMW-AN----------T-SSP-------------------------------SVSP------ : 20
 : ---------------------------------------------------------------------MAMEEE-----------------GDMW-AN----------A-TSP-------------------------------SPSP------ : 20
 : ----------------------------------------------------------------------------------------------------------------------------------------------------------- : -
 : ---------------------------------------------------------------------MAMEHE-----------------EDMW-AN----------T-TSP-------------------------------SASP------ : 20
 : ---------------------------------------------------------------------MAMEDE-----------------EDMW-AN----------T-TNP-------------------------------SASP------ : 20
 : ---------------------------------------------------------------------MAMEDE-----------------EDMW-AN----------T-SSP-------------------------------SVSP------ : 20
 : ---------------------------------------------------------------------MAMEDE-----------------EGMW-AN----------T-TSP-------------------------------SASP------ : 20
 : MA------------------------------------------------------------------MAAMEDD-----------------EDMW-AV----------T-TSP-------------------------------SASP------ : 23
 : ---------------------------------------------------------------------MAMEDD-----------------EDMW-AN----------T-SSP-------------------------------SASP------ : 20
 : MA------------------------------------------------------------------AMDLEDD-----------------EDIW-GK----------TASSP-------------------------------SASP------ : 24
 : MA------------------------------------------------------------------AIDMEDD-----------------DDIW-AN----------TASSP-------------------------------SASP------ : 24
 : MA------------------------------------------------------------------AMDMDDE-----------------EDMW-AN----------T-TSP-------------------------------SASP------ : 23
 : -----------------------------------------------------------------------MEEDD----------------AEIW-GN------------TSP-------------------------------SVSP------ : 18
 : -----------------------------------------------------------------------MGGA-------------------LW------------SAATASVRWH----RSRDHLAHAAGDSISMSMCSCSASLNP------ : 43
 : MR------------------------------------------------------------------AFPLEK-----------------------------------------------------------------------NRAPFTF--- : 15
 : ----------------------------------------------------------------------------------------------------------------------------------------------------------- : -
 : -----------------------------------------------------------------------MERA-----------------AG---------------TGSGG---------------------------DDDELVLP------ : 19
 : -----------------------------------------------------------------------MEKA-------------------------------------------------------------------DDELVSP------ : 11
 : -----------------------------------------------------------------------MEKA-------------------------------------------------------------------DDELVLP------ : 11
 : -----------------------------------------------------------------------MEQA------------------------------------------------------------------DDGRFVSP------ : 12
 : -----------------------------------------------------------------------MDTVK----------------ATVW-GS----------N-TAS-------------------------------QSSP------ : 19
 : -----------------------------------------------------------------------MEE--------------------VW-KD-MSLSTA------TPAAG-----LYHLRLQRHP-------------PLPP------ : 32
 : -----------------------------------------------------------------------MEE--------------------VW-KD-MSLSTA------TPAAG-----LYPLRLQRHH-------------PPSP-----S : 33
 : -----------------------------------------------------------------------MED--------------------LW-KE-MSL----------PAAT-----FYHLHLQRHP-----------DSYPSP-----S : 31
 : -----------------------------------------------------------------------MEE--------------------VW-RDYMSLQST---CATTPAVH---------------------------SSPSPPSSYRD : 33
 : -----------------------------------------------------------------------MEEE------------------VIW-KD-MSSLSS------TPVAG-------------------------------------- : 20
 : -----------------------------------------------------------------------MEE--------------------VW-KDYMSLQPS--LISMTPAAGGGGALYSHRHHQHHLSPS------GAGASHSPPSF--- : 52
 : M--------------------------STNKVSSNSPPKSL-YTCSSPSTLSSPSPIPNQS-----TTGNSMEE--------------------VW-ND-INLASL------------------HEHPNSHTGS---------NNNTDDHVF--H : 72
 : MWSS-------------PGANIDNNNTSNSKVSGNSPSKCFSSTCSSPSPPSPSPPIPNQS-----MNGASMEE--------------------VW-DD-INLASL------------------HDHSNTNTSS-----------NTNHHSF--N : 84
 : MLSSAKHQRNHRLS----ATNKNQTLTKVSSISSSSPSSS-----SSSSSTSSSSPLPSQDSQAQKRSLVTMEE--------------------VW-ND-INLASIHHLNRHSP------------HPQHN--HEPRFR-GQNHHNQNP------ : 103
 : -----------------------------------------------------------------------MEE--------------------VW-KE-INLGSL------------------HYHRQLNIGHEPMLK------NQNP-----N : 33
 : MQIASFYPPDSRLRSLGPGSPVHE-MWSVEMNKNGAPALS--STSSSSSSTLHSPPRSNRKT---------MED--------------------VW-RD-LNLTLL-------P------------------------------RDEGPKGF--R : 82
 p
 160 * 180 * 200 * 220 * 240 * 260 * 280 * 300 *
 : --------------------------------------------------QLS------LSGC------------------------SSL-----------FSI-ANSSTGG---------GAGAHANNVA-----------------ARLQQLP : 47
 : --------------------------------------------------RLS------LSGC------------------------SSL-----------FSI-SSSSSTG--------GGGGARGNVAA-----------------ARLQLPP : 48
 : --------------------------------------------------QLS------LSGC------------------------SSL-----------FSL-SSSSTGG---------GAGAHAN--------------------DVVNAAT : 44
 : --------------------------------------------------QLS------LSGC------------------------SSL-----------FSL-SSSSAAR--------------------------------------LQLP- : 32
 : --------------------------------------------------QLS------LSSC------------------------SSF-----------LSI-SSAGTSA---------ADGA------------------------------ : 35
 : -------------------------------------------------GQLS------LSSF------------------------SSL-----------FSV-SSATSTP--------T---------------------------PHLPPL- : 38
 : --------------------------------------------------QLS------LSGC------------------------SSL-----------FSL-SSTSTSR--------DNDSAAAA------APPP----------SSLHPLP : 44
 : --------------------------------------------------QLS------LSGC------------------------SSL-----------FSL-SSTSTSR--------DNDDSAAALTAPVPAPPAG---------ASLHPLP : 51
 : --------------------------------------------------QLS------LSGC------------------------SSL-----------FSL-SSSSTGR--------DNDGAAAA--------------------PAMPPLP : 40
 : --------------------------------------------------QLSI-----MSGC------------------------SSL-----------FSI-STLRDDD--------DGAAAAVV--------------------LAGHAL- : 58
 : --------------------------------------------------QLSI-----MSGC------------------------SSL-----------FSI-SALRDDD--------DGAAAAVV--------------------LAGHAL- : 59
 : --------------------------------------------------QLSI-----MSGC------------------------SSL-----------FSI-STLPDDD--------DGTAAAFV--------------------LTDRAL- : 58
 : -----------------------------------------------------------MSSC------------------------SSL-----------FSI-STLRDDD--------DD--GAVV--------------------LAGHAL- : 29
 : --------------------------------------------------QLSI-----MSGC------------------------SSL-----------FSI-STLRDDD--------DD--GAVV--------------------LAGHAL- : 57
 : --------------------------------------------------QLSI-----MSGC------------------------SSL-----------FSI-STLRDDD--------DG---AVV--------------------LAGHAL- : 60
 : --------------------------------------------------QLSF-----VSGC------------------------SSL-----------FSI-STLQDDD--------DG---AVV--------------------IAGHAL- : 57
 : --------------------------------------------------QLSF-----VSGC------------------------SSL-----------FSI-STLQDDD--------DDGRPAVV--------------------IAGHAM- : 62
 : --------------------------------------------------QLSF-----VSGC------------------------SSL-----------FSI-STLQDDD--------DG--DAVV--------------------IAGHAL- : 57
 : --------------------------------------------------QLSI-----MSGC------------------------SSL-----------FSI-STLRDDD--------DG--GGVR--------------------LAGAAL- : 60
 : --------------------------------------------------QLSI-----MSGC------------------------SSL-----------FSI-STLRDDD--------DG--GGVR--------------------LAGAAL- : 60
 : --------------------------------------------------QLSI-----MSGC------------------------SSL-----------FSI-STLRDDD--------DG--GGVR--------------------LAGAAL- : 60
 : --------------------------------------------------QLSL-----MSGC------------------------SSL-----------FSI-SVLRDGD--------DLGGG-------------------------VRSL- : 51
 : --------------------------------------------------QLS------LSGC------------------------SSL-----------FTI-SAGSGSL--------DTASPT------------------------VRGPP : 44
 : -------------PT-PAPAASTC---------------------SFIFTALS------PNSSR-VH-LLYSG--------------SSP------------PH-SSDNNYNRGM-GSD--GGGGYRNAAASPA--------------SFFSSGG : 88
 : -------------LT-PAAAASTC---------------------SFISTALS------LNSSH-LH-LLYSG--------------ASP------------PH-SSDNNYNGGM-GCDGDGGGGYRNAAASPP--------------S-FSSAD : 89
 : ------------------------------------------------------------------------------------------------------------------M-GC-----AGYRNAVASPA--------------SFFSSAG : 21
 : -------------PTAAAAAASTC---------------------SFISTALS------LNSSH-LQ-LLYSG--------------VSP------------PH-SSDNNYDGGI-SCN---GGGYRNAVASPA--------------SFFCA-G : 87
 : -------------PT-PAAAASTC---------------------SLISTALS------LNSSH-LH-LLYSG--------------ASP------------SH-SSDNNYNGSM-GCD----GGYRNAAASPA--------------PFLSS-- : 84
 : -------------PT-SAAAASTC---------------------SFISTALS------LNSSH-LH-LLYSG--------------ASP------------SH-SSDNNYSGSM-GCD----GGYRNAAASPA--------------PFFSSAG : 86
 : -------------PT---PAASTC---------------------SFISTALS------LRSS---H-LLYSGAS------------PSP------------SH-SSDNNYNRSM-GCD--GRGGYSNAAAS-----------------LFSSAG : 83
 : -------------PA-TAAA-------------------------AAISTALS------LNPR--LH-LLASS--------------SSP------------FH---------------PGFGGCSRNAAAASP--------------PCFFSAA : 75
 : -------------PR----------------------------PRGFISTALS------LNSTH-LQGLLPSSFVDA---------AASP------------CH-ASGNN----------NGGGDGRNAAPMSS--------------IFFASAS : 81
 : ------------TPL-TTAVVAPC--------------------GAFISTQLS------LNSR--LQ-LLSTTAAAG---------GSSP-PHSVGA----GIF-AADGL-RHHV-GLGDGGGGGFRNAPASPA--------------PFFSAYG : 106
 : -------------PQ-PVAAASTC--------------------SAFISTQLS------LNSR--LH-LLSSAAAAG---------GSSPTTRGVGA----GAY-AADGV-RHHM-SL--GVGGGFRNAVASPA--------------SSFFPYN : 104
 : -------------PQ-PGA----C---------------------SFISTQLS------LNSR--LH-LLSSSAA------------SPPDHCGGGGGGDGGIY-GADGGFDHHH-LM--GGGGGFRNAASVSP--------------GSFFPYG : 100
 : -------------PA-PHPA-PPC---------------------SFISTALS------LNSS---H-LLAGAH-------------DAP------------AYYSSGDN---------DAAAADPSASAVSPA--------------SLFSSS- : 78
 : -------------PR------------------------RNN--KHPNAYHLRPRLQLCLDALPVPH-MLVSHHQPR---------GSSQ---GTAQRREEVRLPSMDGAYDELV--------SSHSHSSGGGT--------------ACLCSAR : 124
 : -------------PD---------K---------------KNRVPSPAGYLLAHW--PCLAGCREVK-GLDSSASRSGQHGGRAKRGMEP---------------ERGDAAELLV----WGTTGAGNNDDGAAA--------------ACLCPA- : 96
 : ---------------------------------------------------------------------------------------MEP---------------QRGE--EQLVRGGGAGCGGGGANDDAGAA--------------ACLCP-- : 35
 : -------------PA-----------------------------------SFQD----GLPSS--------------------------------------RSY-PSC------------IGGGS------------------------------ : 41
 : -------------PA-AHATTSTT--------------------DMPISTALS------LSAT------------------------SSY------------SH-SSGDT----------------TNNAVVGI--------------ACLCP-- : 57
 : -------------PA-AYATTITA--------------------AMPISTALS------LTAS------------------------SSP-----------SSY--SGYT---------------TNNNDVVGV--------------ACLCP-- : 58
 : -------------PA-AHNAT-TT--------------------AMPISTALS------LTAS------------------------SSR------------IY-TSNDVVG-------------VANDS----F-------------LSSCP-- : 57
 : -----------NPPF-AVAA------------------------AASISTALS------LTSP--------------------------------------QSY-SSDTN---------------NDDAAIRSA--------------SNLYAY- : 63
 : ----------SSAPMPQ------------------------PLPLTLPPTALS------LSST------------------------LE------------FTNL----------------GAGPPSNSSSGDDTRGDLMPDPFAGEQTDIFSF- : 94
 : ANIVVLQDFLASAPVPQPPTPPPP-----------------PLPLPLPPTALS------LSST------------------------LE------------FTYLSAG------------AGAGPPSNSSSGDDSRGDFMPDSYAGARTDIFSFA : 117
 : ANI------LVSASVPP---------------------------PPLPPTALSL----SLSST------------------------LE------------FTYL-----------------GAGASNSSSGDDSRG----DPFA---GDIFSF- : 88
 : AGI--LQDFFTSTPQQP------------------------QTPPPLPPTVLS------LNFT------------------------LE------------FTFL-----------------GSGSSSTSSGDPA-------------TVIFPY- : 89
 : ---L-QQHYYLSSPARS------------------------CFHRPPPPTALA------LSST------------------------LE------------FTYLGCA--------------AATASNSSSGDDAQ--LMP-PAADLDDILISF- : 87
 : -----------SSPL----------------------------QPPTPTTTLS------LSSN-----------------------TLE------------FTYLGGAGAGA--------GSGGGGGSSTSGDDSR-----------DLMVFSF- : 107
 : GMM--FQDLLARSSNKDTPTRVASK---EPSSGGGNNFLKNS-LGPPPATMLN------LNYG-------------------------------------------------------------------------------------------- : 123
 : GMV--FQDFLARPSNKDTSTRAASK---EPSSGGGNSFLKNS-LGPPPATMLS------LNSG------------------------SDH-----------FHYLESSNTVPV------------RPNPQMHSHANGGT---------ISFDSSL : 171
 : NSI--FQDFLKGSLNQEPAPTSQTTGSAPNGDSTTVTVLYSS-PFPPPATVLS------LNSG------------------------AG------------FEFLDNQDPLV-------------TSNSNLHTHHHLS----NAHAFNTSFEAL- : 195
 : NSI--FQDFLNMPLNQPPPP------PPPPSSSTIVTALYGSLPLPPPATVLS------LNSG------------------------VG------------FEFLDTTENLL-------------ASNP-------------------RSFEES- : 105
 : EMI--LQDFLTTGNDTRRAQQDEIH---VERRGG---------PQNVAATNLS------LITG------------------------SD------------LHYLDWLSYKP-------------ASAVQEEEQQRPP----------HVLEPGA : 158
 ls
 Gu.an_033932.1
 Bo.am_047054.1
 Bo.am_000187.1
 Ol.la_007769.1
 Or.sa_02g58670.1
 Br.di_3g60870.1
 Se.it_3G230700.1
 So.bi_004G359200.1
 Ot.th_03274A
 Bo.am_013110.2
 Phy.ed_24830.t1
 Ol.la_004263.1
 Bo.am_019707.1
 Gu.an_001152.1
 Phy.ed_31929.t1
 Se.it_4G282900.1
 So.bi_010G269000.1
 Ot.th_16409A
 Or.sa_06g50600.1
 Or.sa_06g50830.1
 Or.sa_06g50480.1
 Br.di_1g29920.1
 Pha.la_02g34110.1
 Phy.ed_05093.t1
 Bo.am_027362.1
 Phy.ed_14669.t1
 Ol.la_037713.1
 Bo.am_035833.1
 Gu.an_019842.1
 Bo.am_038479.1
 Br.di_4g36587.1
 Or.sa_09g36910.1
 Se.it_2G291300.1
 So.bi_002G280800.1
 Ot.th_06520A
 Pha.la_09g16690.1
 Ol.la_016238.1
 Se.it_6G240100.1
 So.bi_007G176700.2
 Or.sa_08g43600.1
 Bo.am_029860.1
 Phy.ed_24093.t2
 Bo.am_035296.1
 Pha.la_08g01350.1
 Bo.am_004836.1
 Phy.ed_47757.t2
 Ol.la_031099.1
 Ot.th_25696A
 So.bi_004G001100.1
 Br.di_3g00300.1
 Po.tr_002G018400.1
 Po.tr_005G243400.1
 Ar.th_4G35900.1
 Ar.th_2G17770.1
 Am.tr_scaffold00152.26
 Gu.an_033932.1
 Bo.am_047054.1
 Bo.am_000187.1
 Ol.la_007769.1
 Or.sa_02g58670.1
 Br.di_3g60870.1
 Se.it_3G230700.1
 So.bi_004G359200.1
 Ot.th_03274A
 Bo.am_013110.2
 Phy.ed_24830.t1
 Ol.la_004263.1
 Bo.am_019707.1
 Gu.an_001152.1
 Phy.ed_31929.t1
 Se.it_4G282900.1
 So.bi_010G269000.1
 Ot.th_16409A
 Or.sa_06g50600.1
 Or.sa_06g50830.1
 Or.sa_06g50480.1
 Br.di_1g29920.1
 Pha.la_02g34110.1
 Phy.ed_05093.t1
 Bo.am_027362.1
 Phy.ed_14669.t1
 Ol.la_037713.1
 Bo.am_035833.1
 Gu.an_019842.1
 Bo.am_038479.1
 Br.di_4g36587.1
 Or.sa_09g36910.1
 Se.it_2G291300.1
 So.bi_002G280800.1
 Ot.th_06520A
 Pha.la_09g16690.1
 Ol.la_016238.1
 Se.it_6G240100.1
 So.bi_007G176700.2
 Or.sa_08g43600.1
 Bo.am_029860.1
 Phy.ed_24093.t2
 Bo.am_035296.1
 Pha.la_08g01350.1
 Bo.am_004836.1
 Phy.ed_47757.t2
 Ol.la_031099.1
 Ot.th_25696A
 So.bi_004G001100.1
 Br.di_3g00300.1
 Po.tr_002G018400.1
 Po.tr_005G243400.1
 Ar.th_4G35900.1
 Ar.th_2G17770.1
 Am.tr_scaffold00152.26
Figure S2 Protein sequence alignment of the FD family members in bamboo and its related species.

## Slide 3
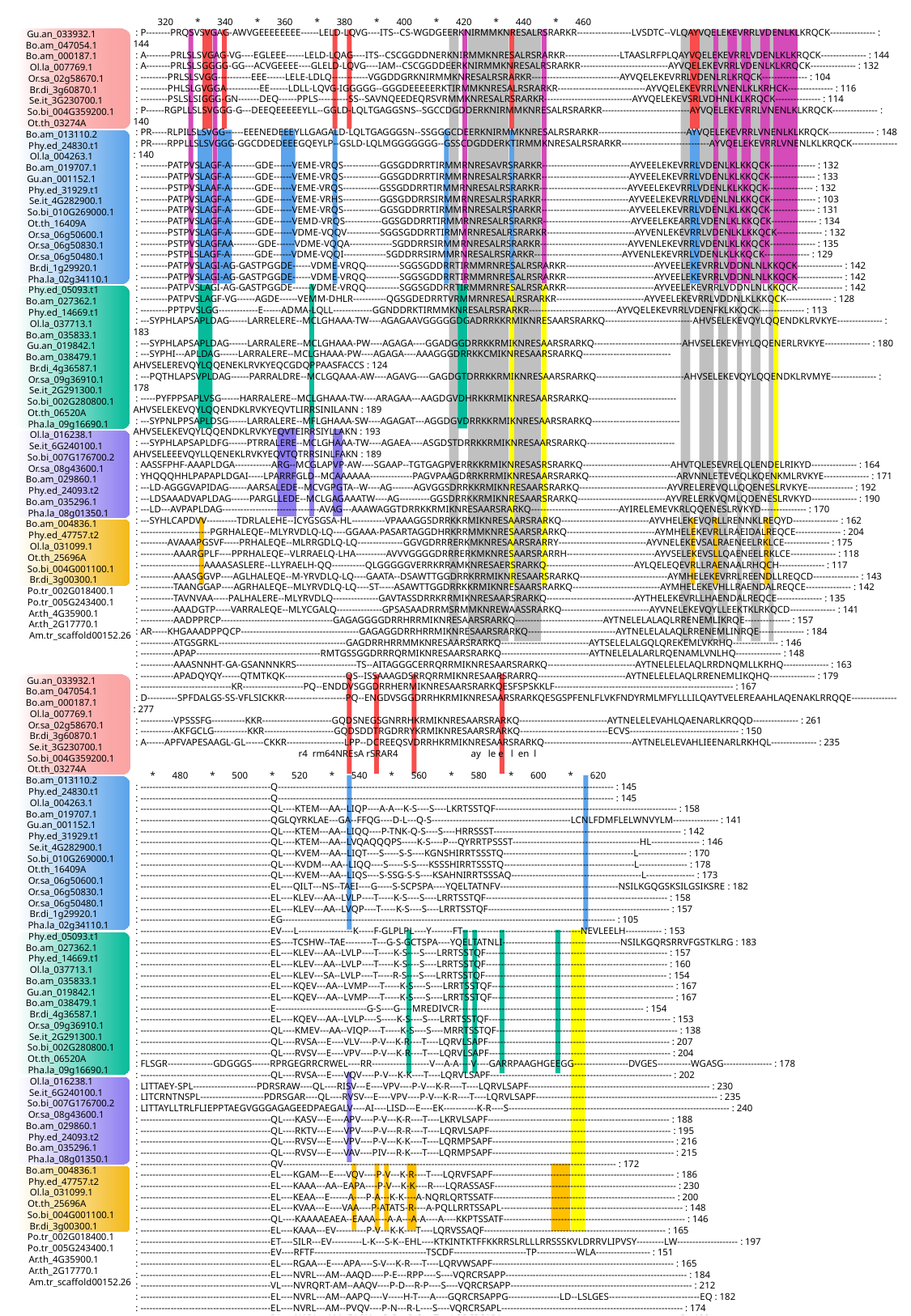

320 * 340 * 360 * 380 * 400 * 420 * 440 * 460
 : P--------PRQSVSVGAG-AWVGEEEEEEEE------LELD-LQVG----ITS--CS-WGDGEERKNIRMMKNRESALRSRARKR------------------LVSDTC--VLQAYVQELEKEVRRLVDENLKLKRQCK--------------- : 144
 : A--------PRLSLSVGAG-VG----EGLEEE------LELD-LQAG----ITS--CSCGGDDNERKNIRMMKNRESALRSRARKR------------------LTAASLRFPLQAYVQELEKEVRRLVDENLKLKRQCK--------------- : 144
 : A--------PRLSLSGGGG-GG---ACVGEEEE----GLELD-LQVG----IAM--CSCGGDDEERKNIRMMKNRESALRSRARKR-----------------------------AYVQELEKEVRRLVDENLKLKRQCK--------------- : 132
 : ---------PRLSLSVGG-----------EEE------LELE-LDLQ------------VGGDDGRKNIRMMKNRESALRSRARKR-----------------------------AYVQELEKEVRRLVDENLRLKRQCK--------------- : 104
 : ---------PHLSLGVGGA-----------EE------LDLL-LQVG-IGGGGG--GGGDEEEEERKTIRMMKNRESALRSRARKR-----------------------------AYVQELEKEVRRLVNENLKLKRHCK--------------- : 116
 : ---------PSLSLSIGGG-GN-------DEQ------PPLS----------SS--SAVNQEEDEQRSVRMMKNRESALRSRARKR-----------------------------AYVQELEKEVSRLVDHNLKLKRQCK--------------- : 114
 : P------RGPLLSLSVGGG-G---DEEQEEEEEYLL--GGLD-LQLTGAGGSNS--SGCCDGDDERKNIRMMKNRESALRSRARKR-----------------------------AYVQELEKEVRRLVNENLKLKRQCK--------------- : 140
 : PR-----RLPILSLSVGG------EEENEDEEEYLLGAGALD-LQLTGAGGGSN--SSGGGCDEERKNIRMMKNRESALRSRARKR-----------------------------AYVQELEKEVRRLVNENLKLKRQCK--------------- : 148
 : PR-----RPPLLSLSVGGG-GGCDDEDEEEGQEYLP--GSLD-LQLMGGGGGGG--GSSCDGDDERKTIRMMKNRESALRSRARKR-----------------------------AYVQELEKEVRRLVNENLKLKRQCK--------------- : 140
 : ---------PATPVSLAGF-A--------GDE------VEME-VRQS------------GGSGDDRRTIRMMRNRESAVRSRARKR-----------------------------AYVEELEKEVRRLVDENLKLKKQCK--------------- : 132
 : ---------PATPVSLAGF-A--------GDE------VEME-VRQS------------GGSGDDRRTIRMMRNRESALRSRARKR-----------------------------AYVEELEKEVRRLVDENLKLKKQCK--------------- : 133
 : ---------PSTPVSLAAF-A--------GDE------VEME-VRQS------------GSSGDDRRTIRMMRNRESALRSRARKR-----------------------------AYVEELEKEVRRLVDENLKLKKQCK--------------- : 132
 : ---------PATPVSLAGF-A--------GDE------VEME-VRHS------------GGSGDDRRSIRMMRNRESALRSRARKR-----------------------------AYVEELEKEVRRLVDENLNLKKQCK--------------- : 103
 : ---------PATPVSLAGF-A--------GDE------VEME-VRQS------------GGSGDDRRTIRMMRNRESALRSRARKR-----------------------------AYVEELEKEVRRLVDENLKLKKQCK--------------- : 131
 : ---------PATPVSLAGF-A--------GDE------VEMD-VRQS------------GGSGDDRRTIRMMRNRESALRSRARKR-----------------------------AYVEELEKEARRLVDENLKLKKQCK--------------- : 134
 : ---------PSTPVSLAGF-A--------GDE------VDME-VQQV-----------SGGSGDDRRTIRMMRNRESALRSRARKR-----------------------------AYVENLEKEVRRLVDENLKLKKQCK--------------- : 132
 : ---------PSTPVSLAGFAA--------GDE------VDME-VQQA--------------SGDDRRSIRMMRNRESALRSRARKR-----------------------------AYVENLEKEVRRLVDENLKLKKQCK--------------- : 135
 : ---------PSTPLSLAGF-A--------GDE------VDME-VQQI--------------SGDDRRSIRMMRNRESALRSRARKR-----------------------------AYVENLEKEVRRLVDENLKLKKQCK--------------- : 129
 : ---------PATPVSLAGI-AG-GASTPGGDE------VDME-VRQQ-----------SGGSGDDRRTIRMMRNRESALRSRARKR-----------------------------AYVEELEKEVRRLVDDNLNLKKQCK--------------- : 142
 : ---------PATPVSLAGI-AG-GASTPGGDE------VDME-VRQQ-----------SGGSGDDRRTIRMMRNRESALRSRARKR-----------------------------AYVEELEKEVRRLVDDNLNLKKQCK--------------- : 142
 : ---------PATPVSLAGI-AG-GASTPGGDE------VDME-VRQQ-----------SGGSGDDRRTIRMMRNRESALRSRARKR-----------------------------AYVEELEKEVRRLVDDNLNLKKQCK--------------- : 142
 : ---------PATPVSLAGF-VG------AGDE------VEMM-DHLR-----------QGSGDEDRRTVRMMRNRESALRSRARKR-----------------------------AYVEELEKEVRRLVDDNLKLKKQCK--------------- : 128
 : ---------PPTPVSLGG-------------E------ADMA-LQLL-------------GGNDDRKTIRMMKNRESALRSRARKR-----------------------------AYVQELEKEVRRLVDENFKLKKQCK--------------- : 113
 : ---SYPHLAPSAPLDAG------LARRELERE--MCLGHAAA-TW----AGAGAAVGGGGGDGADRRKKRMIKNRESAARSRARKQ-----------------------------AHVSELEKEVQYLQQENDKLRVKYE--------------- : 183
 : ---SYPHLAPSAPLDAG------LARRALERE--MCLGHAAA-PW----AGAGA----GGADGGDRRKKRMIKNRESAARSRARKQ-----------------------------AHVSELEKEVHYLQQENERLRVKYE--------------- : 180
 : ---SYPHI---APLDAG------LARRALERE--MCLGHAAA-PW----AGAGA----AAAGGGDRRKKCMIKNRESAARSRARKQ-----------------------------AHVSELEREVQYLQQENEKLRVKYEQCGDQPPAASFACCS : 124
 : ---PQTHLAPSVPLDAG------PARRALDRE--MCLGQAAA-AW----AGAVG----GAGDGTDRRKKRMIKNRESAARSRARKQ-----------------------------AHVSELEKEVQYLQQENDKLRVMYE--------------- : 178
 : -----PYFPPSAPLVSG------HARRALERE--MCLGHAAA-TW----ARAGAA---AAGDGVDHRKKRMIKNRESAARSRARKQ-----------------------------AHVSELEKEVQYLQQENDKLRVKYEQVTLIRRSINILANN : 189
 : ---SYPNLPPSAPLDSG------LARRALERE--MFLGHAAA-SW----AGAGAT---AGGDGVDRRKKRMIKNRESAARSRARKQ-----------------------------AHVSELEKEVQYLQQENDKLRVKYEQVTEIRRSIYLLAKN : 193
 : ---SYPHLAPSAPLDFG------PTRRALERE--MCLGHAAA-TW----AGAEA----ASGDSTDRRKKRMIKNRESAARSRARKQ-----------------------------AHVSELEEEVQYLLQENEKLRVKYEQVTQTRRSINLFAKN : 189
 : AASSFPHF-AAAPLDGA------------ARG--MCGLAPVP-AW----SGAAP--TGTGAGPVERRKKRMIKNRESASRSRARKQ-----------------------------AHVTQLESEVRELQLENDELRIKYD--------------- : 164
 : YHQQQHHLPAPAPLDGAI-----LPARRFGLD--MCAAAAAA--------------PAGVPAAGDRRKRRMIKNRESAARSRARKQ-----------------------------ARVNNLETEVEQLKQENKMLRVKYE--------------- : 171
 : ---LD-AGGGVAPIDAG------AARSALEDE--MCVGPGTA--W----AG-------AGVGGSDRRKKRMIKNRESAARSRARKQ-----------------------------AYVRELEREVQLLQQENESLRVKYE--------------- : 192
 : ---LDSAAADVAPLDAG------PARGLLEDE--MCLGAGAAATW----AG----------GGSDRRKKRMIKNRESAARSRARKQ-----------------------------AYVRELERKVQMLQDENESLRVKYD--------------- : 190
 : ---LD---AVPAPLDAG--------------------------------AVAG---AAAWAGGTDRRKKRMIKNRESAARSRARKQ-----------------------------AYIRELEMEVKRLQQENESLRVKYD--------------- : 170
 : ---SYHLCAPDVV----------TDRLALEHE--ICYGSGSA-HL-----------VPAAAGGSDRRKKRMIKNRESAARSRARKQ-----------------------------AYVHELEKEVQRLLRENNKLREQYD--------------- : 162
 : -----------------------PGRHALEQE--MLYRVDLQ-LQ----GGAAA-PASARTAGGDHRKRRMMKNRESAARSRARKQ-----------------------------AYMHELEKEVRLLRAEIDALREQCE--------------- : 204
 : ---------AVAAAPGSVF-----PRHALEQE--MLRRGDLQ-LQ---------------GGVGDRRRERKMKNRESAARSRARRY-----------------------------AYVNELEKEVSALRAENEELRKLCE--------------- : 175
 : -----------AAARGPLF----PPRHALEQE--VLRRAELQ-LHA----------AVVVGGGGDRRRERKMKNRESAARSRARRH-----------------------------AYVSELEKEVSLLQAENEELRKLCE--------------- : 118
 : ---------------------AAAASASLERE--LLYRAELH-QQ-----------QLGGGGGVERRKRRAMKNRESAERSRARKQ-----------------------------AYLQELEQEVRLLRAENAALRHQCH--------------- : 117
 : -----------AAASGGVP----AGLHALEQE--M-YRVDLQ-LQ----GAATA--DSAWTTGGDRRKRRMIKNRESAARSRARKQ-----------------------------AYMHELEKEVRRLREENDLLREQCD--------------- : 143
 : -----------TAANGGAP----AGRHALEQE--MLYRVDLQ-LQ----ST-----ASAWTTGGDRRKKRMIKNRESAARSRARKQ-----------------------------AYMHELEKEVHLLRAENDALREQCE--------------- : 142
 : -----------TAVNVAA-----PALHALERE--MLYRVDLQ---------------GAVTASSDRRKRRMIKNRESAARSRARKQ-----------------------------AYTHELEKEVRLLHAENDALREQCE--------------- : 135
 : -----------AAADGTP-----VARRALEQE--MLYCGALQ---------------GPSASAADRRMSRMMKNREWAASSRARKQ-----------------------------AYVNELEKEVQYLLEEKTKLRKQCD--------------- : 141
 : -----------AADPPRCP-------------------------------------GAGAGGGGDRRHRRMIKNRESAARSRARKQ-----------------------------AYTNELELALAQLRRENEMLIKRQE--------------- : 157
 : AR-----KHGAAADPPQCP---------------------------------------GAGAGGDRRHRRMIKNRESAARSRARKQ-----------------------------AYTNELELALAQLRRENEMLINRQE--------------- : 184
 : -----------ATGSGRKL------------------------------------------GAGDRRHRRMMKNRESAARSRARKQ-----------------------------AYTSELELALGQLQREKEMLVKRHQ--------------- : 146
 : -----------APAP-----------------------------------------RMTGSSGGDRRRQRMIKNRESAARSRARKQ-----------------------------AYTNELELALARLRQENAMLVNLHQ--------------- : 148
 : -----------AAASNNHT-GA-GSANNNKRS--------------------TS--AITAGGGCERRQRRMIKNRESAARSRARKQ-----------------------------AYTNELELELAQLRRDNQMLLKRHQ--------------- : 163
 : -----------APADQYQY------QTMTKQK--------------------QS--ISSAAAGDSRRQRRMIKNRESAARSRARRQ-----------------------------AYTNELELELAQLRRENEMLIKQHQ--------------- : 179
 : ------------------------------KR--------------------PQ--ENDDVSGGDRRHERMIKNRESAARSRARKQESFSPSKKLF----------------------------------------------------------- : 167
 : D----------SPFDALGS-SS-VFLSICKKR--------------------PQ--ENGDVSGGDRRHKRMIKNRESAARSRARKQESGSPFENLFLVKFNDYRMLMFYLLLILQAYTVELEREAAHLAQENAKLRRQQE--------------- : 277
 : -----------VPSSSFG-----------KKR-----------------------GQDSNEGSGNRRHKRMIKNRESAARSRARKQ-----------------------------AYTNELELEVAHLQAENARLKRQQD--------------- : 261
 : -----------AKFGCLG-----------KKR-----------------------GQDSDDTRGDRRYKRMIKNRESAARSRARKQ-----------------------------ECVS------------------------------------ : 150
 : A------APFVAPESAAGL-GL------CKKR-------------------LPP--DCREEQSVDRRHKRMIKNRESAARSRARKQ-----------------------------AYTNELELEVAHLIEENARLRKHQL--------------- : 235
 r4 rm64NREsA rSRAR4 ay le e l en l
 * 480 * 500 * 520 * 540 * 560 * 580 * 600 * 620
 : -------------------------------------------Q--------------------------------------------------------------------------------------------------------------- : 145
 : -------------------------------------------Q--------------------------------------------------------------------------------------------------------------- : 145
 : -------------------------------------------QL----KTEM---AA--LIQP----A-A---K-S----S----LKRTSSTQF------------------------------------------------------------ : 158
 : -------------------------------------------QGLQYRKLAE---GA--FFQG----D-L---Q-S----------------------------------------------LCNLFDMFLELWNVYLM--------------- : 141
 : -------------------------------------------QL----KTEM---AA--LIQQ----P-TNK-Q-S----S----HRRSSST-------------------------------------------------------------- : 142
 : -------------------------------------------QL----KTEM---AA--LVQAQQQPS-----K-S----P---QYRRTPSSST------------------------------------------HL---------------- : 146
 : -------------------------------------------QL----KVEM---AA--LIQT----S-----S-S----KGNSHIRRTSSSTQ-------------------------------------------L---------------- : 170
 : -------------------------------------------QL----KVDM---AA--LIQQ----S-----S-S----KSSSHIRRTSSSTQ-------------------------------------------L---------------- : 178
 : -------------------------------------------QL----KVEM---AA--LIQS----S-SSG-S-S----KSAHNIRRTSSSAQ-------------------------------------------L---------------- : 173
 : -------------------------------------------EL----QILT---NS--TAEI----G-----S-SCPSPA----YQELTATNFV---------------------------------------NSILKGQGSKSILGSIKSRE : 182
 : -------------------------------------------EL----KLEV---AA--LVLP----T-----K-S----S----LRRTSSTQF------------------------------------------------------------ : 158
 : -------------------------------------------EL----KLEV---AA--LVQP----T-----K-S----S----LRRTSSTQF------------------------------------------------------------ : 157
 : -------------------------------------------EG-------------------------------------------------------------------------------------------------------------- : 105
 : -------------------------------------------EV----L------------------K-----F-GLPLPL----Y-------FT---------------------------------------NEVLEELH------------ : 153
 : -------------------------------------------ES----TCSHW--TAE---------T---G-S-GCTSPA----YQELTATNLI---------------------------------------NSILKGQRSRRVFGSTKLRG : 183
 : -------------------------------------------EL----KLEV---AA--LVLP----T-----K-S----S----LRRTSSTQF------------------------------------------------------------ : 157
 : -------------------------------------------EL----KLEV---AA--LVLP----T-----K-S----S----LRRTSSTQF------------------------------------------------------------ : 160
 : -------------------------------------------EL----KLEV---SA--LVLP----T-----R-S----S----LRRTSSTQF------------------------------------------------------------ : 154
 : -------------------------------------------EL----KQEV---AA--LVMP----T-----K-S----S----LRRTSSTQF------------------------------------------------------------ : 167
 : -------------------------------------------EL----KQEV---AA--LVMP----T-----K-S----S----LRRTSSTQF------------------------------------------------------------ : 167
 : -------------------------------------------E------------------------------G-S----G----MREDIVCR------------------------------------------------------------- : 154
 : -------------------------------------------EL----KQEV---AA--LVLP----S-----K-S----S----LRRTSSTQF------------------------------------------------------------ : 153
 : -------------------------------------------QL----KMEV---AA--VIQP----T-----K-S----S----MRRTSSTQF------------------------------------------------------------ : 138
 : -------------------------------------------QL----RVSA---E----VLV----P-V---K-R----T----LQRVLSAPF------------------------------------------------------------ : 207
 : -------------------------------------------QL----RVSV---E----VPV----P-V---K-R----T----LQRVLSAPF------------------------------------------------------------ : 204
 : FLSGR---------------GDGGGS------RPRGEGRRCRWEL----RR-------------------V---A-A----V----GARRPAAGHGEEGG------------------DVGES-----------WGASG---------------- : 178
 : -------------------------------------------QL----RVSA---E----VQV----P-V---K-K----T----LQRVLSAPF------------------------------------------------------------ : 202
 : LITTAEY-SPL---------------------PDRSRAW----QL----RISV---E----VPV----P-V---K-R----T----LQRVLSAPF------------------------------------------------------------ : 230
 : LITCRNTNSPL---------------------PDRSGAR----QL----RVSV---E----VPV----P-V---K-R----T----LQRVLSAPF------------------------------------------------------------ : 235
 : LITTAYLLTRLFLIEPPTAEGVGGGAGAGEEDPAEGALV----AI----LISD---E----EK-----------K-R----S------------------------------------------------------------------------- : 240
 : -------------------------------------------QL----KASV---E----APV----P-V---K-R----T----LKRVLSAPF------------------------------------------------------------ : 188
 : -------------------------------------------QL----RKTV---E----VPV----P-V---R-R----T----LQRVLSAPF------------------------------------------------------------ : 195
 : -------------------------------------------QL----RVSV---E----VPV----P-V---K-K----T----LQRMPSAPF------------------------------------------------------------ : 216
 : -------------------------------------------QL----RVSV---E----VAV----PIV---R-K----T----LQRMPSAPF------------------------------------------------------------ : 215
 : -------------------------------------------QV-------------------------------------------------------------------------------------------------------------- : 172
 : -------------------------------------------EL----KGAM---E----VQV----P-V---K-R----T----LQRVFSAPF------------------------------------------------------------ : 186
 : -------------------------------------------EL----KAAA---AA--EAPA----P-V---K-K----R----LQRASSASF------------------------------------------------------------ : 230
 : -------------------------------------------EL----KEAA---E------A----P-A---K-K----A-NQRLQRTSSATF------------------------------------------------------------ : 200
 : -------------------------------------------EL----KVAA---E----VAA----P-ATATS-R----A-PQLLRRTSSAPL------------------------------------------------------------ : 148
 : -------------------------------------------QL----KAAAAEAEA--EAAA----A-A---A-A----A----KKPTSSATF------------------------------------------------------------ : 146
 : -------------------------------------------EL----KAAA---EV----------P-V---K-K----T----LQRVSSAQF------------------------------------------------------------ : 165
 : -------------------------------------------ET----SILR---EV----------L-K---S-K--EHL----KTKINTKTFFKKRRSLRLLLRRSSSKVLDRRVLIPVSY---------LW-------------------- : 197
 : -------------------------------------------EV----RFTF-------------------------------------TSCDF------------------------TP-------------WLA------------------ : 151
 : -------------------------------------------EL----RGAA---E----APA----S-V---K-R----T----LQRVWSAPF------------------------------------------------------------ : 165
 : -------------------------------------------EL----NVRL---AM--AAQD----P-E---RPP----S----VQRCRSAPP------------------------------------------------------------ : 184
 : -------------------------------------------VL----NVRQRT-AM--AAQV----P-D---R-P----S----VQRCRSAPP------------------------------------------------------------ : 212
 : -------------------------------------------EL----NVRL---AM--AAPQ----V-----H-T----A----GQRCRSAPPG-----------------LD--LSLGES------------------------------EQ : 182
 : -------------------------------------------EL----NVRL---AM--PVQV----P-N---R-L----S----VQRCRSAPL------------------------------------------------------------ : 174
 : -------------------------------------------DL----NARL---AM--EAQV----P-D---R-S----T----LQRCRSAPA-----------------------------------------------------------P : 190
 : -------------------------------------------EL----NLRL---AMSSSAQV----P-AHYSS-T----S----LQRCRSAPP------------------------------------------------------------ : 210
 : ----------------------------------------------------------------------------------------------------------------------------------------------------------- : -
 : -------------------------------------------RF----LAAA-------PAQL----P-K---K-N----T----LYRTSTAPF------------------------------------------------------------ : 301
 : -------------------------------------------QL----KMAA---A----IQQ----P-K---K-N----T----LQRSSTAPF------------------------------------------------------------ : 285
 : --------------------------------------------------------------------P-----H----------------SSTF------------------------------------------------------------ : 156
 : -------------------------------------------TF----ANPT---AA--FQHGK---T-----KPA----T----LRRSTTAPF------------------------------------------------------------ : 262
 Gu.an_033932.1
 Bo.am_047054.1
 Bo.am_000187.1
 Ol.la_007769.1
 Or.sa_02g58670.1
 Br.di_3g60870.1
 Se.it_3G230700.1
 So.bi_004G359200.1
 Ot.th_03274A
 Bo.am_013110.2
 Phy.ed_24830.t1
 Ol.la_004263.1
 Bo.am_019707.1
 Gu.an_001152.1
 Phy.ed_31929.t1
 Se.it_4G282900.1
 So.bi_010G269000.1
 Ot.th_16409A
 Or.sa_06g50600.1
 Or.sa_06g50830.1
 Or.sa_06g50480.1
 Br.di_1g29920.1
 Pha.la_02g34110.1
 Phy.ed_05093.t1
 Bo.am_027362.1
 Phy.ed_14669.t1
 Ol.la_037713.1
 Bo.am_035833.1
 Gu.an_019842.1
 Bo.am_038479.1
 Br.di_4g36587.1
 Or.sa_09g36910.1
 Se.it_2G291300.1
 So.bi_002G280800.1
 Ot.th_06520A
 Pha.la_09g16690.1
 Ol.la_016238.1
 Se.it_6G240100.1
 So.bi_007G176700.2
 Or.sa_08g43600.1
 Bo.am_029860.1
 Phy.ed_24093.t2
 Bo.am_035296.1
 Pha.la_08g01350.1
 Bo.am_004836.1
 Phy.ed_47757.t2
 Ol.la_031099.1
 Ot.th_25696A
 So.bi_004G001100.1
 Br.di_3g00300.1
 Po.tr_002G018400.1
 Po.tr_005G243400.1
 Ar.th_4G35900.1
 Ar.th_2G17770.1
 Am.tr_scaffold00152.26
 Gu.an_033932.1
 Bo.am_047054.1
 Bo.am_000187.1
 Ol.la_007769.1
 Or.sa_02g58670.1
 Br.di_3g60870.1
 Se.it_3G230700.1
 So.bi_004G359200.1
 Ot.th_03274A
 Bo.am_013110.2
 Phy.ed_24830.t1
 Ol.la_004263.1
 Bo.am_019707.1
 Gu.an_001152.1
 Phy.ed_31929.t1
 Se.it_4G282900.1
 So.bi_010G269000.1
 Ot.th_16409A
 Or.sa_06g50600.1
 Or.sa_06g50830.1
 Or.sa_06g50480.1
 Br.di_1g29920.1
 Pha.la_02g34110.1
 Phy.ed_05093.t1
 Bo.am_027362.1
 Phy.ed_14669.t1
 Ol.la_037713.1
 Bo.am_035833.1
 Gu.an_019842.1
 Bo.am_038479.1
 Br.di_4g36587.1
 Or.sa_09g36910.1
 Se.it_2G291300.1
 So.bi_002G280800.1
 Ot.th_06520A
 Pha.la_09g16690.1
 Ol.la_016238.1
 Se.it_6G240100.1
 So.bi_007G176700.2
 Or.sa_08g43600.1
 Bo.am_029860.1
 Phy.ed_24093.t2
 Bo.am_035296.1
 Pha.la_08g01350.1
 Bo.am_004836.1
 Phy.ed_47757.t2
 Ol.la_031099.1
 Ot.th_25696A
 So.bi_004G001100.1
 Br.di_3g00300.1
 Po.tr_002G018400.1
 Po.tr_005G243400.1
 Ar.th_4G35900.1
 Ar.th_2G17770.1
 Am.tr_scaffold00152.26
Figure S2 Protein sequence alignment of the FD family members in bamboo and its related species.

## Slide 4
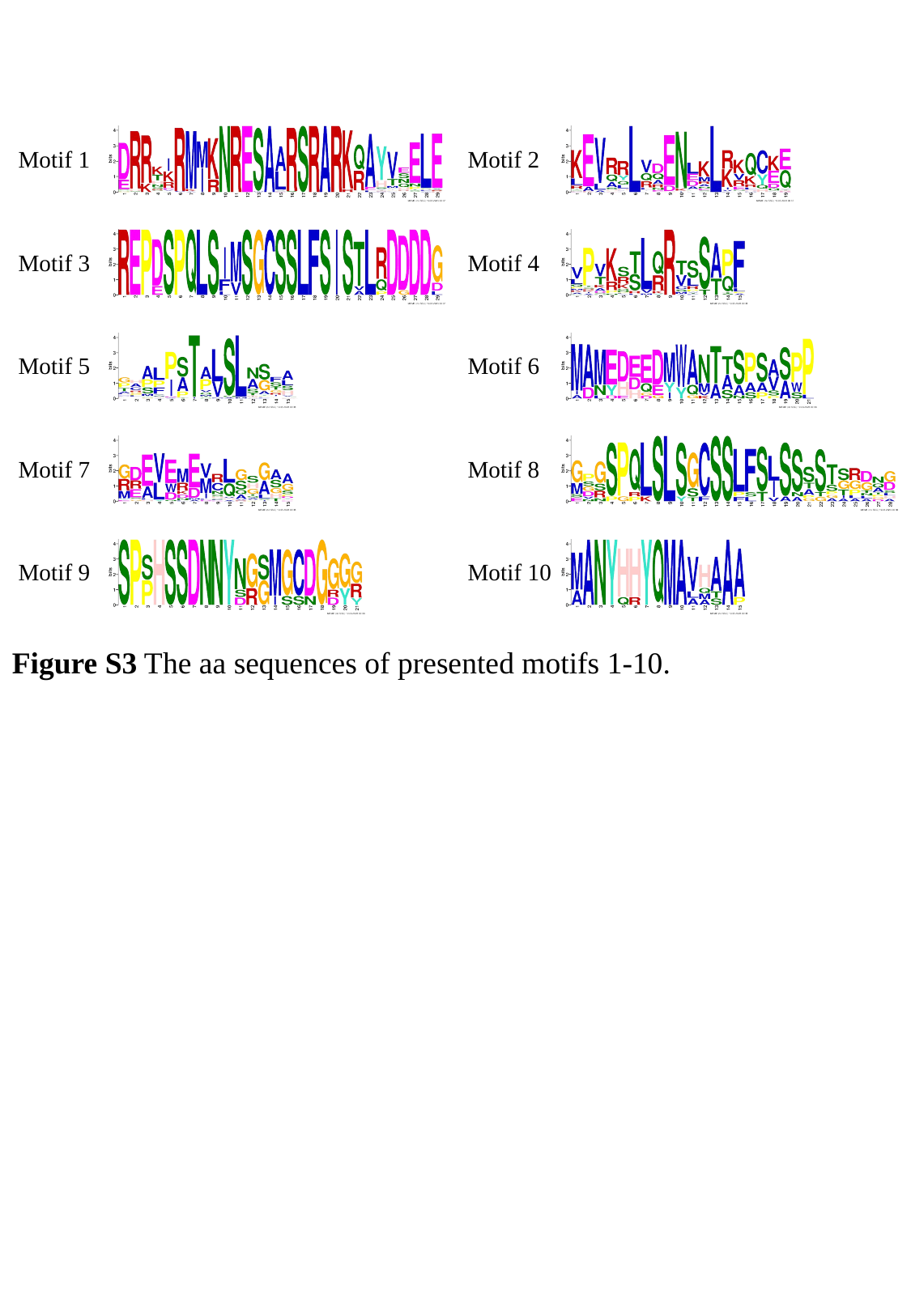

Motif 1
Motif 2
Motif 3
Motif 4
Motif 5
Motif 6
Motif 7
Motif 8
Motif 9
Motif 10
Figure S3 The aa sequences of presented motifs 1-10.
